# Temporal Framing of Thalamic Relay-Mode Firing by Phasic Inhibition during the Alpha Rhythm

**DOI:** 10.1016/j.neuron.2009.08.012

**Published:** 2009-09-10

**Authors:** Magor L. Lőrincz, Katalin A. Kékesi, Gábor Juhász, Vincenzo Crunelli, Stuart W. Hughes

**Affiliations:** 1 School of Biosciences, Cardiff University, Museum Avenue, Cardiff CF10 3AX, UK; 2 Department of Physiology and Neurobiology, Eötvös Loránd University, Pázmány P. st. 1/C, 1117 Budapest, Hungary; 3 Laboratory of Proteomics, Institute of Biology, Eötvös Loránd University, Pázmány P. st. 1/C, 1117 Budapest, Hungary

**Keywords:** SYSNEURO

## Abstract

Several aspects of perception, particularly those pertaining to vision, are closely linked to the occipital alpha (α) rhythm. However, how the α rhythm relates to the activity of neurons that convey primary visual information is unknown. Here we show that in behaving cats, thalamocortical neurons in the lateral geniculate nucleus (LGN) that operate in a conventional relay-mode form two groups where the cumulative firing is subject to a cyclic suppression that is centered on the negative α rhythm peak in one group and on the positive peak in the other. This leads to an effective temporal framing of relay-mode output and results from phasic inhibition from LGN interneurons, which in turn are rhythmically excited by thalamocortical neurons that exhibit high-threshold bursts. These results provide a potential cellular substrate for linking the α rhythm to perception and further underscore the central role of inhibition in controlling spike timing during cognitively relevant brain oscillations.

## Introduction

The EEG alpha (α) (8–13 Hz) rhythm is intimately associated with many basic aspects of perception (VanRullen and Koch, 2003; Fingelkurts and Fingelkurts, 2006; Mathewson et al., 2009). For example, both reaction time (Surwillo, 1961) and the maximal interstimulus interval for perceived simultaneity (Kristofferson, 1967) are highly correlated with α rhythm frequency. In the specific case of vision, the ability to accurately perceive certain events (Varela et al., 1981), or even to perceive them at all (Nunn and Osselton, 1974; Busch et al. 2009; Mathewson et al., 2009), has been reported to be dependent on the particular phase of the α rhythm at which they occur. This has led to the suggestion that the α rhythm provides “excitability cycles” that act to temporally frame or gate perceptual events (Bartley, 1940; Lindsley, 1952; Lansing, 1957; Wiener, 1985; Crick and Koch, 2003; VanRullen and Koch, 2003; Fingelkurts and Fingelkurts, 2006; Mathewson et al., 2009) and which may ultimately provide a viable basis for discrete perceptual processing in the brain, i.e., the concept that perception, particularly pertaining to vision, occurs in discrete snapshots or processing epochs lasting around 70–100 ms (Stroud, 1955; Efron, 1970). Surprisingly, while the idea that the α rhythm provides a temporal framework for perception has often been discussed and promoted, cellular-level evidence of a link between spontaneous α activity and the firing of neurons thought to be involved in perceptual processing is currently lacking.

A key brain area in both the transmission of visual information and the generation of the α rhythm is the primary visual thalamus or dorsal lateral geniculate nucleus (LGN) (da Silva et al., 1973; Chatila et al., 1993; Rougeul-Buser and Buser, 1997; Hughes et al., 2004; Hughes and Crunelli, 2005). In this structure, a specialized subset (∼25%–30%) of thalamocortical (TC) neurons exhibit intrinsic rhythmic burst firing at α frequencies, termed high-threshold (HT) bursting, which occurs coherently with naturally occurring α waves in vivo (Hughes et al., 2004; Hughes and Crunelli, 2005) and which can be synchronized by gap junctions (GJs), i.e., electrical synapses, to form an α rhythm pacemaker unit (Hughes et al., 2004; Hughes and Crunelli, 2005; Lőrincz et al., 2008). While the strong intrinsic rhythmicity of these cells is ideally suited to driving thalamic and cortical α oscillations (Llinás, 1988), it is generally accepted that the faithful transmission of visual information from the retina to the neocortex is carried out by the conventional single spike or so-called relay-mode of firing that occurs in the remainder and overwhelming majority of LGN TC neurons (Llinás and Jahnsen, 1982). However, the precise temporal association between activity in relay-mode TC neurons and the α rhythm is unknown.

In cortical circuits the timing of principal cell firing during cognitively relevant brain oscillations is largely determined by the coordinated activity of various types of inhibitory interneurons (Klausberger and Somogyi, 2008). Recently, we hypothesized that an engagement of local inhibitory cells may also be a key component in phasing the output of relay-mode TC neurons in the LGN during natural α activity (Hughes and Crunelli, 2005). In the current study we therefore investigated how relay-mode LGN TC neurons and thalamic inhibitory neurons, i.e., LGN interneurons and neurons of the perigeniculate nucleus (PGN), the visual sector of the thalamic reticular nucleus (TRN), are engaged during α rhythms. To do this, we combined simultaneous recordings of the occipital EEG, the LGN local field potential (LFP), and LGN and PGN unit activity during natural wakefulness in behaving cats with mechanistic experiments in an LGN-PGN slice preparation where the capacity to generate network α activity is preserved (Lőrincz et al., 2008). Using this approach we demonstrate that the firing of relay-mode LGN TC neurons is intimately linked to ongoing α activity, with these cells forming two groups that show a transient suppression of firing close to the positive and negative α rhythm peaks. This scenario results from phasic inhibition from LGN interneurons, effectively translates to a temporal framing of relay-mode output, and may ultimately provide a candidate cellular-level basis for explaining the well known link between visual perception and the α rhythm.

## Results

### The Activity of Both HT Bursting and Relay-Mode LGN TC Neurons Is Correlated with the α Rhythm

**Figure 1:**
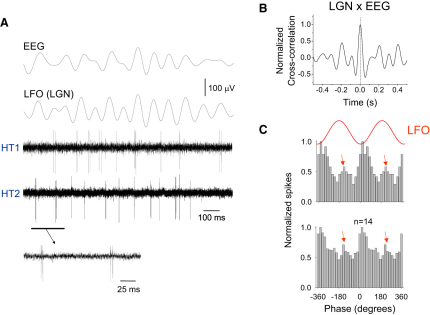
Naturally Occurring α Rhythms Are Related to Synchronized HT Bursting in a Subset of LGN TC Neurons (A) Simultaneous occipital EEG recording and recordings of local field oscillations (LFO) and TC neuron activity from the ipsilateral LGN during an α rhythm epoch in a behaving cat. The two unit recordings are from HT bursting TC neurons (underlined section is expanded below). (B) Cross-correlogram of the LGN and EEG recordings shown in (A) over an extended recording period (see also Figure S1). (C) (Top) Spike timing histogram for the neuron labeled HT2 (in A), constructed from 14 consecutive α rhythm epochs, indicates that while most spikes occur close to the negative LFO peak, a significant group (red arrows) also occurs near the positive peak. (Bottom) Cumulative spike timing histogram for 14 different HT bursting TC neurons shows a similar general form.

During natural wakefulness EEG recordings in freely moving cats regularly exhibited α rhythm episodes (duration: 7.1 ± 2.9 s; frequency: 8.9 ± 1.2 Hz; n = 75 episodes; α rhythm density: 24.02% ± 1.48%) that were synchronized with local field oscillations (LFOs) in the LGN (Figures 1A and 1B; see also Figure S1 available online) (da Silva et al., 1973; Chatila et al., 1993; Hughes et al., 2004). As noted previously (Hughes et al., 2004), during these episodes, single-unit recordings from a subset of TC neurons (n = 14 of 42; 33%) showed rhythmic HT bursts (interspike interval [ISI]: 11.2 ± 4.8 ms; spikes per burst: 2.8 ± 0.9; n = 1400 bursts from 14 neurons) (Figure 1A). All HT bursting TC neurons were significantly correlated with the LFO (p < 0.01; Rao's test) with the majority of spikes occurring close to the negative peak (21.5° ± 8.0°; n = 14) (Figure 1C), but with a substantial cluster also occurring near the positive LFO peak (196.5° ± 9.4°; n = 14) (Figure 1C, red arrows), thus matching results from previous in vitro investigations of these cells (Hughes et al., 2004; Lőrincz et al., 2008).

**Figure 2:**
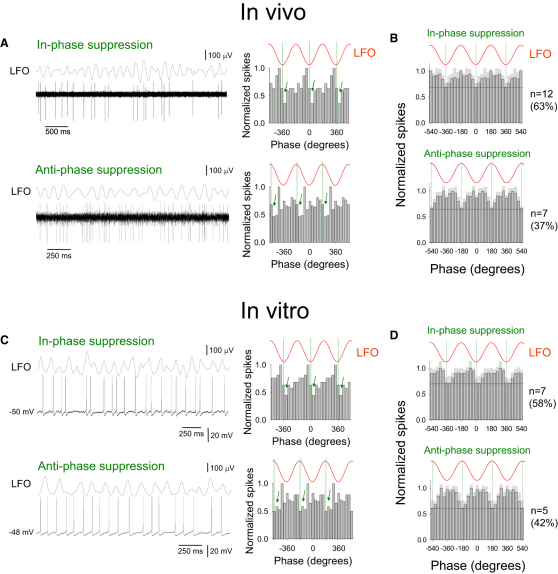
Relay-Mode Firing in LGN TC Neurons In Vivo and In Vitro Is Suppressed near the Negative or Positive Peak of the α Rhythm (A) LGN recordings, from a behaving cat, of the α rhythm and two tonic firing, i.e., relay-mode, TC neurons where the minimum amount of firing (green arrows) occurs close to the negative (top, in-phase suppression) and positive (bottom, antiphase suppression) LFO peaks. Corresponding spike timing histograms are on the right. (B) (Top) Cumulative spike timing histogram for 12 relay-mode TC neurons where minimum firing (dotted line) occurred close to the negative LFO peak (i.e., in-phase suppression). Note how this essentially translates to a cyclic facilitation centered on the positive LFO peak. (Bottom) Cumulative spike timing histogram for seven relay-mode TC neurons where minimum firing (dotted line) occurred close to the positive LFO peak (i.e., antiphase suppression), in this case essentially translating to a cyclic facilitation centered on the negative LFO peak. The light gray shaded area is the standard error. (C) Simultaneous in vitro LGN recordings of the Cch-induced α rhythm and two relay-mode TC neurons where the minimum amount of firing (green arrows) occurs close to the negative (top, in-phase suppression) and positive (bottom, antiphase suppression) LFO peaks. Note the similarity of the corresponding spike timing histograms, shown to the right, with those produced from the in vivo data. (D) (Top) Cumulative spike timing histogram for seven spontaneously active relay-mode TC neurons recorded in vitro where minimum firing occurred close to the negative LFO peak (i.e., in-phase suppression) has a similar form to that obtained from in vivo data. (Bottom) The cumulative spike timing histogram for five spontaneously active relay-mode TC neurons recorded in vitro where minimum firing occurred near the positive LFO peak (i.e., antiphase suppression) is also similar to its in vivo counterpart. Note: 50 μM Cch was present for all in vitro experiments depicted in this and subsequent figures.

In the remaining 67% of TC neurons (n = 28 of 42), we observed single spikes only, i.e., tonic or relay-mode firing (Figure 2A). In most of these cells (n = 19 of 28; 67%) this firing was also significantly correlated with α activity (p < 0.05; Rao's test). However, unlike HT bursting TC neurons, relay-mode TC neurons did not show a reproducible, stereotypical pattern of action potential output with respect to the LFO and there was not a consistent phase of the LFO at which they preferred to generate action potentials (Figure S2B, left). On the other hand, the phases of the oscillation cycle at which the minimum amount of firing occurred in relay-mode TC neurons were clustered around either the negative LFO peak (mean phase: 15.1° ± 17.5°; n = 12 of 19; 63%) (in-phase suppression, green arrows, Figure 2A, top; Figure S2B, right, black bars) or the positive LFO peak (mean phase: 177.8° ± 15.8°; n = 7 of 19; 37%) (antiphase suppression, green arrows, Figure 2A, bottom; Figure S2B, right, white bars).

To gain an insight into the overall output that would be generated by ensembles of α-rhythm-correlated relay-mode TC neurons, we constructed cumulative spike timing histograms, with respect to the LFO, for the two groups of these cells. As would be expected given that neurons in each group share a common phase at which the minimum amount of firing occurs but show no communal point in the oscillation cycle where maximum firing takes place (Figure S2B), these histograms exhibited an essentially flat profile apart from a suppression of firing near the negative (Figure 2B, top) or positive (Figure 2B, bottom) LFO peaks, respectively. For the group of relay-mode TC neurons exhibiting an in-phase suppression of firing, at its minimum point this firing was suppressed to 68.8% ± 8.7% (n = 12) of the maximum value, whereas for relay-mode TC neurons that were subject to antiphase suppression, firing was diminished to 65.6% ± 13.3% (n = 7) (black dashed lines, Figure 2B, top and bottom). Thus, a cyclic suppression of firing in relay-mode TC neurons effectively leads to a temporal framing of output from these cells (see also Supplemental Results Section B).

### Firing in LGN Interneurons Is Also Correlated with α Rhythms

**Figure 3:**
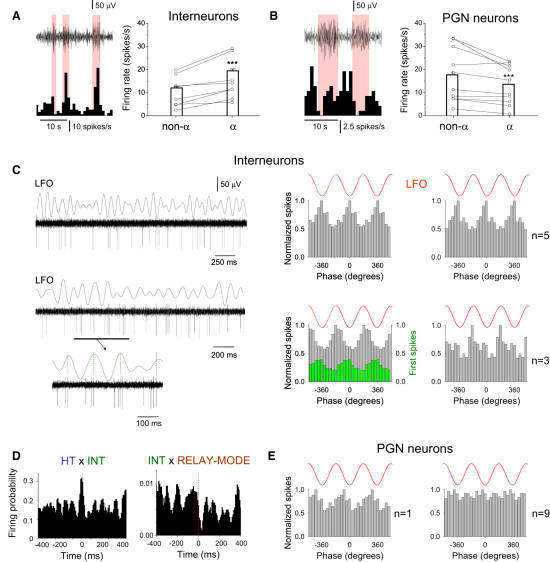
Acitivity of Putative LGN Interneurons and PGN Neurons during α Activity in Naturally Waking Cats (A) (Left) LFP recording from the LGN bandpass filtered at 5–15 Hz (top) and corresponding firing rate histogram of a putative LGN interneuron (bottom). Note how periods of higher-amplitude α oscillations (light red bars) are accompanied by increased firing. (Right) Summary of firing rate changes in eight LGN interneurons during and outside α rhythm epochs (see Experimental Procedures). (B) (Left) LFP recording from the LGN bandpass filtered at 5–15 Hz (top) and corresponding firing rate histogram of a PGN neuron (bottom). Higher-amplitude α oscillations (light red bars) are correlated with decreased firing. (Right) Summary of firing rate changes in 10 PGN neurons during and outside α rhythm epochs. (C) (Top) Unit activity of a putative LGN interneuron recorded in vivo simultaneously with the LFO. The spike timing histogram for 10 consecutive α rhythm epochs is shown to the immediate right and reveals a preference for firing close to the negative LFO peak. The cumulative spike timing histogram for five distinct cells of this type is shown to the far right. (Bottom) Unit activity of a different LGN interneuron, again recorded in vivo along with the LFO. The underlined section (enlarged below) reveals unusual bursts where the first ISI is notably longer than subsequent ISIs. The spike timing histogram for 10 consecutive α rhythm epochs is shown to the immediate right (gray bars) and reveals that this cell preferentially fires near the positive LFO peak. This is due to the presence of prolonged bursts because the first spikes in a burst (green bars) occur preferentially near the negative LFO peak. The cumulative spike timing histogram for three distinct cells of this type is shown to the far right. (D) (Left) Cross-correlogram for an HT bursting TC neuron-interneuron pair in the LGN reveals a strong correlation between the firing of the two cells. (Right) Cross-correlogram for an interneuron-relay-mode TC neuron pair reveals that interneuron firing is related to a strong suppression of relay-mode firing (see also Figure S4). (E) (Left) Spike timing histogram for the only PGN neuron that was correlated with α activity. (Right) Cumulative spike timing histogram for the other nine PGN neurons recorded in vivo. Error bars represent mean ± SEM.

To test our proposal that inhibitory thalamic neurons play a key role in shaping the output of TC neuron firing during α rhythms (Hughes and Crunelli, 2005), we analyzed the activity of putative LGN interneurons in vivo (n = 8; see Experimental Procedures and Figure S3). During naturally occurring α rhythms, LGN interneurons exhibited an increase in firing rate (during α: 17.35 ± 0.73 Hz; non-α: 10.89 ± 0.54 Hz; n = 8 neurons; p < 0.001) (Figure 3A) and otherwise displayed firing properties that were quite distinct from those of either HT bursting or relay-mode TC neurons (Figures 3C and S3A). In particular, they generated spike bursts that exhibited a significantly larger mean ISI than those presented by HT bursting TC neurons (18.9 ± 4.3 ms; n = 400 bursts from 4 neurons; p < 0.01) (Figure 3C). Spike timing histograms revealed that while all interneurons were significantly correlated with α activity (p < 0.05; Rao's test; n = 8), for five of these neurons, action potential output preferentially occurred close to the negative LFO peak (11.2° ± 4.7°; p < 0.05) (Figure 3C, top), whereas in the remaining three neurons, action potentials were generated primarily close to the positive LFO peak (168.2° ± 17°; p < 0.05) (Figure 3C, bottom). Close inspection of the latter group of interneurons revealed that this could be ascribed to the presence of prolonged bursts (up to eight spikes) that often displayed an unusually long ISI between the first and second spike (35.5 ± 1.6 ms; n = 30 bursts) (Figure 3C, bottom, enlarged section). Simultaneous recordings of interneurons with HT bursting TC neurons often revealed clear synchrony between the two types of cells (n = 5 pairs) (Figure 3D, left; see also Figure S4A), whereas firing in interneurons could be associated with a suppression of activity in relay-mode TC neurons (n = 4 pairs) (Figure 3D, right; see also Figure S4B).

### PGN Neuron Firing Is Reduced during α Rhythm Epochs and Is Largely Uncorrelated with α Activity

During low-frequency (∼7–12 Hz) cortical oscillations in the somatosensory system of rats that occur in quiet waking and immobility and which are often considered to be the analog of the human mu (μ) rhythm in this species (i.e., the somatosensory equivalent of the classical α rhythm), neurons in the TRN generate robust, rhythmic bursting in register with individual LFP waves (reviewed in Hughes and Crunelli, 2005). Rhythmic bursting in TRN neurons has also been shown to play a key role in some types of sleep-related (Domich et al., 1986) and anesthesia-related (Hughes and Crunelli, 2005) oscillations. To investigate whether TRN neurons are involved in α activity during natural wakefulness in cats, we obtained unit recordings from the PGN while concurrently monitoring the LFP in the neighboring LGN. We found that unlike LGN interneurons, the firing rate of PGN neurons was significantly reduced during α rhythm episodes (during α: 13.53 ± 0.79 Hz; non-α: 17.61 ± 1.11 Hz; n = 10 neurons; p < 0.001) (Figure 3B). In addition, spike timing histograms revealed that only one of these cells was significantly correlated with α activity (p < 0.05; Rao's test), firing preferentially just before the negative LFO peak, (Figure 3E, left), whereas the remaining majority (n = 9) were not (p > 0.05; Rao's test) (Figure 3E, right).

### Activity of Relay-Mode TC Neurons Recorded In Vitro during Cholinergically Induced α Rhythms Is Equivalent to that Observed In Vivo

Our previous work has suggested that LGN α rhythms and related TC neuron firing require thalamic activation of muscarinic acetylcholine receptors (mAChRs) (Lőrincz et al., 2008) and/or metabotropic glutamate receptor 1a (mGluR1a) (Hughes et al., 2004). In vivo reverse microdialysis experiments confirmed a role for these receptors but also showed that LGN α rhythms are considerably more susceptible to blocking mAChRs than to antagonizing mGluR1a, indicating that they are mainly reliant on a cholinergic drive (Supplemental Results Section A, Tables S1 and S2 available online, and Figure S5). Consistent with this, intracellular recordings of relay-mode LGN TC neurons, obtained in vitro during α rhythms that had been induced by reinstating the cholinergic drive with the nonspecific AChR agonist carbachol (Cch) (50 μM) (Lőrincz et al., 2008), revealed a pattern of action potential output almost identical to that observed in vivo (i.e., Figures 2A and 2B and Figure S2B). Out of a total of 30 relay-mode TC neurons, 21 (70%) displayed output that was significantly correlated with the LFO (p < 0.05; Rao's test), with 62% (n = 13 of 21) of these showing a minimum rate of firing close to the negative peak (18.1° ± 8.6°) (in-phase suppression, green arrows, Figure 2C, top) and 38% (n = 8 of 21) close to the positive peak (182.2° ± 9.6°) (antiphase suppression, green arrows, Figure 2C, bottom). Of these 21 correlated cells, 12 (58%) exhibited action potential output that was entirely spontaneous (i.e., did not require steady depolarizing current). As this spontaneous firing most closely parallels that which occurs in vivo, only these cells were selected for additional analysis and comparison with in vivo results.

When relay-mode TC neurons recorded in vitro were partitioned into two groups based on whether their minimum firing occurred close to the negative (n = 7 of 12; 58%) or positive (n = 5 of 12; 42%) LFO peak, the cumulative firing in each group was again characterized by histograms that exhibited an essentially flat profile except for a brief suppression of firing near the negative (Figure 2D, top) or positive (Figure 2D, bottom) LFO peaks, respectively. For both the in-phase and antiphase suppressed groups, the average amount by which firing was suppressed at its minimum point was not significantly different to the equivalent values observed in vivo (in-phase suppressed cells: 70.1% ± 13.3%, n = 7, p > 0.5; antiphase suppressed cells: 61.3% ± 15.1%, n = 5, p > 0.5) (black dashed lines, Figure 2D, top and bottom). More importantly, the spike timing histograms pertaining to the two groups of cells were statistically indistinguishable from their in vivo counterparts (Mardia-Watson-Wheeler test: p = 0.72 and p = 0.46, respectively; Watson U^2^ test: p > 0.5 for both cases). Notably, despite the general effects of mGluR activation on TC neurons being similar to those of activating mAChRs, but again consistent with in vivo α rhythms being primarily supported by a cholinergic drive (Supplemental Results Section A, Tables S1 and S2, and Figure S5), pharmacological activation of mGluRs in vitro did not lead to temporal framing in relay-mode TC neurons (Supplemental Results Section H and Figures S14 and S15).

### The Subthreshold Activity of Relay-Mode TC Neurons Recorded In Vitro Is Closely Linked to α Activity

**Figure 4:**
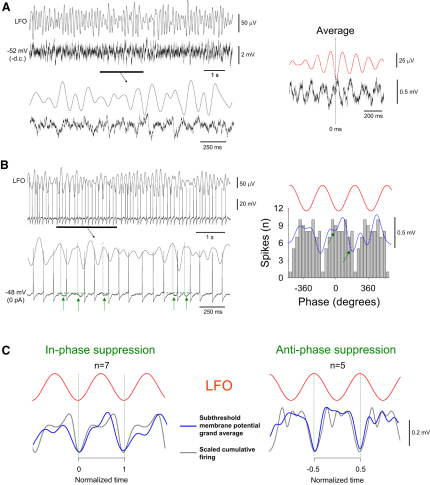
The Output of Relay-Mode TC Neurons In Vitro Is Closely Related to Their α-Rhythm-Derived Subthreshold Membrane Potential Dynamics (A) Simultaneous recording of the subthreshold membrane potential (bottom) in a relay-mode LGN TC neuron in vitro and the proximal LFO (top) (underlined section is expanded below). The LFO-triggered membrane potential average is shown on the right. Note the clear correlation between the LFO and membrane potential. (B) Same cell as in (A) during a brief period of spontaneous relay-mode firing. The underlined section (expanded below) shows the interruption and temporal offsetting of firing by transient hyperpolarizing excursions (green arrows) that are initiated close to the positive LFO peak (vertical dotted lines). The plot on the right shows the corresponding spike timing histogram, which reveals a clear suppression of firing near the positive LFO peak. The blue trace is the membrane potential average shown in (A) filtered at 2–20 Hz. Note the presence of two clear troughs (green arrows) and how action potential timing corresponds closely with subthreshold activity. (C) Grand averages (blue traces) of the time-normalized LFO-triggered subthreshold membrane potential average for the seven in-phase suppressed (left) and five antiphase suppressed (right) cells depicted in Figure 2D. The gray traces are scale-independent spline curves generated from the corresponding cumulative spike timing histograms in Figure 2D. Note the qualitative correspondence between subthreshold activity and action potential output.

Having established that during cholinergically induced in vitro α rhythms the output of spontaneously active relay-mode TC neurons is equivalent to that observed in vivo, we further scrutinized the dynamics of individual relay-mode TC neurons in this condition in order to dissect the cellular events that lead to a differential, cyclic modulation of firing. Hyperpolarization of relay-mode TC neurons below action potential threshold revealed subthreshold membrane potential fluctuations that were phase related to local network activity (n = 12) (Figure 4A). Moreover, in all cases, the relationship between this subthreshold activity and the LFO was qualitatively similar to the relationship between the LFO and action potential timing (Figures 4B, S6A, and S6C). Thus, for in-phase suppressed cells, the LFO-triggered subthreshold membrane potential average was most hyperpolarized close to the negative LFO peak (Figures S6A and S6B) (11.1° ± 4.6°; n = 7), whereas antiphase suppressed cells were on average most hyperpolarized near the positive peak (Figures 4A, S6C, and S6D) (186.6° ± 5.5°; n = 5). When LFO-triggered membrane potential averages were time-normalized and combined together for each group of cells to produce grand averages, the general form of the resulting traces were found to qualitatively match the respective cumulative spike timing histograms (Figure 4C). Interestingly, despite not being evident in the grand average, in individual relay-mode TC neurons that were subject to an antiphase suppression of firing we noted that rather than simply exhibiting a single transient hyperpolarization of membrane potential during each oscillation cycle, the subthreshold membrane potential average showed two clear troughs: a dominant one coincident with the positive LFO peak, but also a smaller one earlier in the cycle (green arrows in Figure 4B; Figures S6C and S6D). This contrasted sharply with the essentially monophasic form of the subthreshold membrane potential averages computed for individual in-phase suppressed cells (Figures S6A and S6B). Notably, those relay-mode TC neurons that did not show spontaneous firing during the LFO (see above) (n = 9) did nonetheless show LFO-correlated subthreshold membrane potential fluctuations. Furthermore, these correlations were of the same form as those exhibited by spontaneously active cells, demonstrating an LFO negative-peak-related hyperpolarization in 67% of cases (n = 6) and a positive-peak-related hyperpolarization in the remainder 33% (n = 3) (data not shown).

### The Subthreshold Activity of Relay-Mode TC Neurons during In Vitro α Rhythms Is Shaped by Phasic Inhibition

Given that the output of relay-mode TC neurons is so closely related to their LFO-related subthreshold dynamics, we asked which mechanisms are central to shaping this activity. We have previously shown that some relay-mode TC neurons exhibit GJ-mediated connections with HT bursting TC neurons (Hughes et al., 2004; Lőrincz et al., 2008). However, these connections are typically weak and appear limited to a minority of cells (Supplemental Results Section C). Also, because HT bursting TC neurons preferentially fire close to the negative LFO peak (Lőrincz et al., 2008), direct GJ coupling with these neurons is unable to explain an in-phase suppression of relay-mode firing, suggesting that additional mechanisms must contribute to shaping the output of relay-mode TC neurons during α rhythms. Indeed, close scrutiny of the subthreshold membrane potential dynamics of relay-mode TC neurons recorded in the presence of Cch revealed occasional small phasic hyperpolarizing events that resembled inhibitory postsynaptic potentials (IPSPs) (amplitude: 1.1 ± 0.1 mV; duration: 47.2 ± 2.9 ms; n = 50 events) (Figure S6; see also Figure 4A). Moreover, for in-phase suppressed cells we observed a preponderance of these events close to the negative LFO peak (Figures S6A and S6B) (3.0° ± 6.4°; n = 7), whereas for antiphase suppressed cells they principally occurred near the positive peak (Figures S6C and S6D; see also Figure 4A) (181.2° ± 26.5°; n = 5). Abolition of these events by the GABA_A_ receptor antagonist SR95531 (i.e., gabazine [GBZ]) (10 μM) (n = 5) confirmed that they were genuine GABAergic IPSPs (Figure S6E).

**Figure 5:**
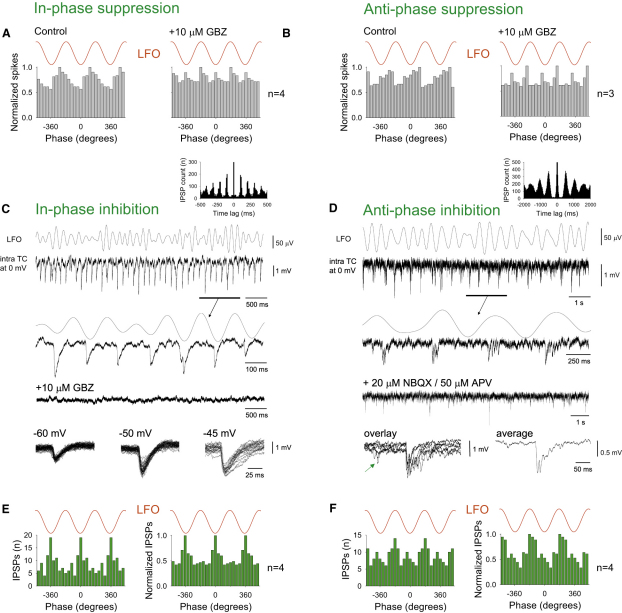
Temporal Framing of Relay-Mode Firing In Vitro Results from GABAergic Inhibition (A) (Left) Cumulative spike timing histograms for four in-phase suppressed relay-mode TC neurons. (Right) This pattern is disrupted by 10 μM GBZ. (B) (Left) Cumulative spike timing histograms for three antiphase suppressed relay-mode TC neurons. (Right) Again, this pattern is disrupted by 10 μM GBZ. (C) Simultaneous recording of an LGN TC neuron at 0 mV with a Cs^+^/QX314-filled electrode and the proximal LFO. The underlined section (enlarged below) shows the presence of rhythmic (see auto-correlogram in the inset above), single (and occasional pairs of) IPSPs that mainly occur in phase with the negative LFO peaks (see E, left). These IPSPs are abolished by 10 μM GBZ. Overlays of several IPSPs (bottom panel) reveal the characteristic voltage-dependent GABA_A_ receptor-mediated IPSPs. (D) Simultaneous recording of a different LGN TC neuron at 0 mV with a Cs^+^/QX314-filled electrode and the proximal LFO. The underlined section (enlarged below) shows the presence of rhythmic (see autocorrelogram in the inset above) IPSP bursts that largely occur in phase with the positive LFO peaks (see F, left). These IPSPs are greatly suppressed by the combined application of NBQX and APV. Overlays of several IPSP bursts (bottom left) reveals that the main burst is often preceded by a single IPSP (green arrow). The average burst is shown to the right. (E) IPSP timing histograms for the TC neuron shown in (C) (left) and for four neurons that showed the same type of phasic inhibition (right). (F) IPSP timing histograms for the TC neuron shown in (D) (left) and for four neurons that showed the same type of phasic inhibition (right). Note: the effects of GBZ on HT bursting TC neurons are described in Supplemental Results Section D and Figure S8.

The presence of IPSPs in relay-mode TC neurons occurring coincidentally with the phase of the LFO at which a suppression of firing occurs suggested that the temporal framing of relay-mode firing is generated by phasic GABAergic inhibition. To test this, in a separate set of experiments we examined the effect of GBZ on action potential timing relative to the LFO in relay-mode TC neurons. In all cases (n = 7), GBZ (10 μM) abolished correlations between relay-mode firing and ongoing α activity (p > 0.05; Rao's test) (Figures 5A and 5B). Despite this result, in most relay-mode TC neurons where we observed a temporal framing of action potential output the presence of unambiguous IPSPs in the subthreshold membrane potential was still surprisingly minimal (i.e., Figure 4 and Figure S6). We reasoned that this was likely to be because while being able to patently influence firing, overt phasic inhibition was largely occluded in the subthreshold membrane potential of these cells by the presence of strong inwardly rectifying K^+^ and other intrinsic conductances that are active near action potential threshold. To test this hypothesis, we made intracellular recordings from TC neurons during Cch-induced α activity with Cs^+^- and QX314-filled electrodes. We found that 76% of relay-mode TC neurons (n = 16 of 21) recorded in this manner exhibited unambiguous IPSPs (amplitude at 0 mV: 1.9 ± 0.24 mV; duration: 25.76 ± 1.13 ms; time to peak: 2.41 ± 0.11 ms; n = 500 events from 10 cells) that (1) occurred with clear rhythmicity (7.88 ± 0.71 Hz; n = 13) (Figures 5C and 5D, insets), (2) were blocked by GBZ (10 μM) (n = 9) (Figure 5C), and (3) displayed a predictable dependence on membrane potential (Figure 5C, bottom traces). Interestingly, in those TC neurons that exhibited prominent IPSPs during recordings with Cs^+^- and QX314-filled electrodes, 63% (n = 10 of 16) displayed mainly one or two events per oscillation cycle that occurred primarily close to the negative LFO peak (5.1° ± 3.9°; p < 0.05; Rao's test; n = 10) (Figures 5C and 5E), whereas the remaining 37% (n = 6 of 16) showed bursts of IPSPs that occurred primarily near the positive LFO peak (185.7° ± 6.7°; p < 0.05; Rao's test; n = 6) (Figures 5D and 5F).

### LGN Interneurons Are the Likely Source of Phasic Inhibition in Relay-Mode TC Neurons

**Figure 6:**
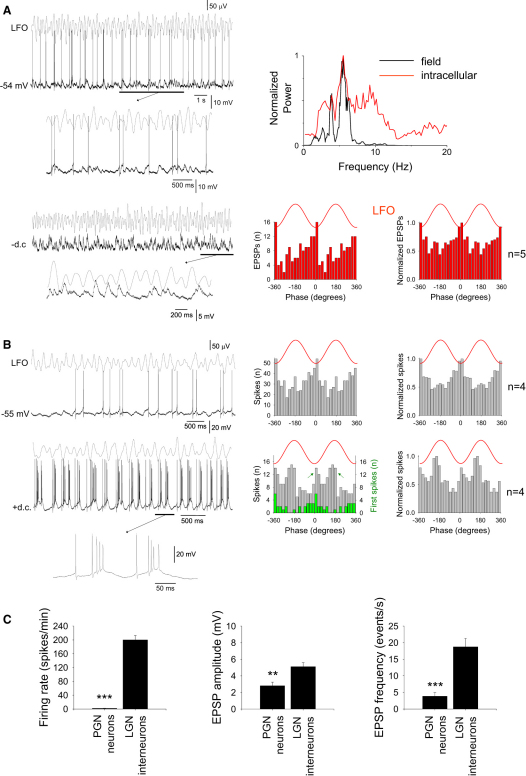
Firing of LGN Interneurons In Vitro Is Correlated with Ongoing α Activity and Can Occur in Two Distinct Modes (A) (Top left) Intracellular recording of an LGN interneuron in the absence of steady current and extracellular recording of the proximal LFO. (Bottom left) Injection of steady hyperpolarizing current exposes prominent excitatory synaptic input (underlined sections are expanded below). (Top right) Power spectra for the LFO (black line) and intracellular subthreshold activity (red line) reveal that the two signals share dominant frequency components. (Bottom right) EPSP timing histograms for the neuron on the left (left plot) and for five different LGN interneurons (right plot) reveal a strong correlation between synaptic events and α activity. (B) (Top) Intracellular recording of another LGN interneuron in the absence of steady current and extracellular recording of the proximal LFO (see also Figure S10B). The corresponding spike timing histogram is shown to the immediate right and a cumulative histogram for four distinct LGN interneurons recorded in the absence of steady current is on the far right. Both plots show that in this condition, firing predominantly occurs near the negative LFO peak. (Bottom) Following injection of 50 pA steady depolarizing current, the cell exhibits rhythmic bursting activity. The underlined section (enlarged below) reveals that individual bursts commonly exhibit a large interval after the first spike. The corresponding spike timing histogram (shown to the immediate right) now exhibits two peaks (green arrows), a small one near the negative LFO peak and a dominant one near the positive peak. The green bars depict the timing of only the first spikes in a burst and demonstrates that although the majority of action potentials in this situation occur near the positive LFO peak, bursts are nonetheless initiated near the negative peak (see also Figure S10A). To the far right is a cumulative spike timing histogram for four distinct LGN interneurons that exhibited similar bursting. (C) Bar graphs summarizing mean firing rate, EPSP amplitude, and individual EPSP frequency in LGN interneurons and PGN neurons recorded in the presence of 50 μM Cch (see also Figure S15C). (^∗∗^p < 0.01; ^∗∗∗^p < 0.001.) Error bars represent mean ± SEM.

**Figure 7:**
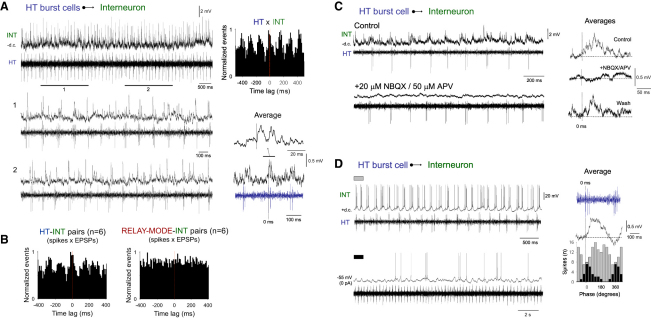
LGN Interneurons Sense and Integrate Activity from the HT Bursting TC Neuron Network In Vitro (A) Subthreshold synaptic activity in an LGN interneuron (INT) and multiunit activity from closely situated HT bursting TC neurons (HT). The sections marked 1 and 2 (expanded below) reveal clear synchrony between HT bursting and synaptic activity in the interneuron. This is confirmed by the discrete cross-correlogram (individual EPSPs × extracellular spikes) shown on the top right and the average subthreshold interneuron activity (triggered by the start of each multiunit burst, shown in blue for reference purposes) shown on the bottom right. (B) Discrete cross-correlograms of the same type as those in A for six HT bursting TC neuron-interneuron pairs (left) and six relay-mode TC neuron-interneuron pairs (right) showing consistent synchronization between the former but not the latter. (C) Subthreshold synaptic activity in an LGN interneuron (INT) and unit activity from a closely situated HT bursting TC neuron (HT) in the presence of 50 μM Cch only (top) and following addition of NBQX and APV (bottom). The average subthreshold interneuron activity (triggered by burst start) for each condition, as well as following NBQX/APV washout, is shown on the right. These reveal that HT bursts are followed by a transient depolarization that is reversibly blocked by NBQX/APV. (D) (Top) Simultaneous intracellular recording of an LGN interneuron depolarized with 30 pA steady current and extracellular recording of a closely situated HT bursting TC neuron. (Bottom) Same recordings but with the interneuron not subject to any steady current. Shown to the right is the average subthreshold activity of the interneuron (black trace; triggered by HT burst start, see blue reference trace). The spike timing histograms for the interneuron relative to HT bursting for the situation where the interneuron is subject to steady depolarization (gray bars) and when steady current is absent (black bars) are shown below. Note that in the absence of depolarizing current, action potentials occur mainly close to onset of the average EPSP, whereas following steady depolarization they predominantly occur after the EPSP peak.

We next turned our attention to identifying the source of phasic inhibition that is present in relay-mode TC neurons during Cch-induced α activity. We reasoned that because these cells receive LFO-correlated rhythmic IPSPs, they must be the recipients of an inhibitory pathway that originates in the rhythmic HT bursting cell network but which must ultimately be administered by either neurons in PGN or local GABAergic interneurons in the LGN (Crunelli et al., 1988). To investigate these two possibilities, we obtained intracellular recordings from both PGN neurons (n = 11) and LGN interneurons (n = 27) (Figure S3) during Cch-induced in vitro α rhythms. These revealed that during α activity both cell types receive barrages of excitatory postsynaptic potential (EPSP) complexes (Figures 6, 7, S9, S10B, and S11). In LGN interneurons these EPSP complexes typically showed overt rhythmicity (6.2 ± 0.7 Hz; n = 14) (Figures S11A and S11B) and were temporally correlated with the negative peak of the LFO in all cells (11.41° ± 5.5°; p < 0.05; Rao's test; n = 14) (Figures 6A and S10B). In PGN neurons, on the other hand, they were essentially nonrhythmic (Figure S9) and were correlated with the LFO (recorded in the LGN) in only 3 out of 11 cells (−51.01° ± 22.1°; p < 0.05; Rao's test; n = 3) (Figures S9B–S9D). The mean amplitude of EPSP complexes in LGN interneurons was 5.1 ± 0.5 mV (n = 200 events from 20 cells), which was significantly larger than the mean amplitude in PGN neurons (2.8 ± 0.5 mV; n = 110 events from 11 cells; p < 0.01) (Figure 6C; see also Figure S15C). Also, the mean frequency of individual EPSPs was significantly greater in LGN interneurons (18.8 ± 2.6 events/s; n = 20) than in PGN neurons (3.8 ± 1.1 events/s; n = 11; p < 0.001) (Figure 6C; see also Figure S15C).

In the absence of any injected steady current, PGN neurons exhibited a very low frequency of action potential firing (action potentials per minute: 1.9 ± 0.3; n = 11) (Figure S9). Extracellular recordings also confirmed a lack of significant action potential output from PGN neurons during α activity (n = 15 slices) (data not shown). In contrast, LGN interneurons displayed considerably more firing (action potentials per minute: 199.9 ± 13.1; n = 20; p < 0.001) (Figure 6). This consisted of a mixture of single action potentials and brief bursts (two to four action potentials; mean: 2.3 ± 0.1; n = 30 events) (Figure 6A, top, and Figure 6B, top) which primarily occurred close to the negative LFO peak (Figure 6B, top right) at a mean angle (5.1° ± 15.8°; n = 4; p < 0.05; Rao's test) that was not significantly different to that at which in-phase IPSPs occur in recordings of relay-mode TC neurons obtained with Cs^+^/QX314-filled electrodes (p > 0.1) (Figure 5C).

Interestingly, when some LGN interneurons (n = 12 of 27; 44%) were slightly more depolarized (≤50 pA), their pattern of firing could be transformed into one consisting of rhythmic bursts of up to 10 action potentials (6.2 ± 0.3; n = 30 events) (Figure 6B, bottom; Figure S11C). These bursts typically exhibited a pronounced delay between the first spike and the remainder of the burst (41.6 ± 2.9 ms; n = 30 events), meaning that the corresponding spike timing histogram displayed a biphasic appearance with a small peak in firing close to the negative LFO peak (7.8° ± 13.1°; n = 5) and a larger one near the positive LFO peak (166.1° ± 12.0°; n = 5) (Figure 6B, bottom right). Importantly, extracellular single-unit recordings of LGN interneurons confirmed that this latter type of activity commonly occurs spontaneously during in vitro α activity (n = 22) (Figure S10A). Furthermore, we found that the mean angle between the two peaks in the corresponding spike timing histograms was not significantly different from the mean angle between the two troughs in the subthreshold membrane potential average of relay-mode TC neurons that are subject to antiphase suppression (161.3° ± 16.9° ms; n = 4; p > 0.5, Watson-Williams F-test) (Figures 4B, S6C, and S6D). Also, for both intracellular and extracellular interneuron recordings, the mean angle at which the antiphase peak occurred was not significantly different to that at which antiphase IPSPs predominantly occurred in recordings of relay-mode TC neurons obtained with Cs^+^/QX314-filled electrodes (p > 0.1) (Figure 5D), and the mean number of spikes per burst (5.5 ± 0.2; n = 200 bursts) was not statistically different from the mean number of IPSPs per burst of IPSPs (4.7 ± 0.2; n = 200 IPSP bursts; p > 0.1).

The two types of firing observed for LGN interneurons in vitro are clearly comparable with the output of putative interneurons recorded during naturally occurring α rhythms in vivo (Figure 3C). Indeed, we found no significant differences in the mean number of spikes per burst between the two scenarios, both for the cases where action potential output preferentially occurred close to the negative LFO peak (in vivo: 2.1 ± 0.2; n = 30 events; p > 0.5) and for the cases where action potentials were primarily generated near the positive LFO peak (in vivo: 5.9 ± 0.6; n = 30 events; p > 0.5). Also, there were no differences in the mean ISIs between the in vitro and in vivo recordings (p > 0.5).

Importantly, in all LGN interneurons recorded in vitro, the combined application of the AMPA/kainate receptor antagonist NBQX (20 μM) and the NMDA receptor antagonist APV (50 μM) not only blocked EPSPs but also eliminated all spontaneous firing (n = 8) (Figures S11A and S11C). This means that if LGN inteneurons are the source of phasic inhibition in relay-mode TC neurons, then blockade of fast glutamatergic synaptic transmission should also suppress IPSPs in these cells. This was indeed the case, with combined NBQX/APV application greatly reducing the quantity of IPSPs in relay-mode TC neurons (control, 50 μM Cch: 8.3 ± 1.2 events/s; NBQX/APV: 0.6 ± 0.3 events/s; n = 4) (Figure 5D). Thus, rhythmic IPSPs and temporal framing in relay-mode TC neurons arise from activity in LGN interneurons that in turn relies on a rhythmic excitatory drive (see also Supplemental Results Section F).

### LGN Interneurons Sense and Integrate Activity from the HT Bursting TC Neuron Network

Because of the synchrony between HT bursting TC neurons and presumed interneurons observed in vivo (Figure 3D), and since EPSPs observed in LGN interneurons during Cch application in vitro exhibit such strong rhythmicity and are temporally correlated with the LFO, we hypothesized that interneurons receive a convergent synaptic excitation from HT bursting TC neurons (see also Supplemental Results Section G). To test this in vitro, we obtained intracellular recordings from interneurons while monitoring the output of closely situated (<200 μm) HT bursting TC neurons with extracellular recordings. In all cases we found that multiunit HT bursting activity was clearly correlated with EPSP generation in LGN interneurons (Figures 7A and 7B). Also, when interneuron activity was averaged over several oscillation cycles, individual HT bursts were associated with a pronounced EPSP complex in interneurons (time from HT burst start to average EPSP complex onset: 3.7 ± 0.8 ms; time to peak: 39.7 ± 6.7 ms; amplitude: 0.7 ± 0.2 mV; n = 7) (Figures 7A, 7C, and 7D), with these average EPSP complexes being reversibly abolished by combined NBQX/APV application (n = 3) (Figure 7C). In contrast, EPSPs in interneurons were never correlated with activity in relay-mode TC neurons (Figure 7B, right).

Finally, although burst firing at depolarized membrane potentials is an intrinsic property of LGN interneurons (Zhu et al., 1999a, 1999b; Pape et al., 1994; Cox et al., 2003; Acuna-Goycolea et al., 2008) (Supplemental Results Section E), the unusual form of individual bursts that are evident in these cells during α activity following the introduction of a small amount of steady current appears to be partly shaped by synaptic input. The reasoning for this comes from the finding that unlike spontaneous rhythmic bursts occurring during network oscillations, bursts elicited by the injection of brief current pulses in the presence of NBQX and APV do not show such a prolonged interval between the first two spikes (12.7 ± 4.7 ms; p < 0.001; n = 6) (Figures S11B and S11D). In fact, individual action potentials during interneuron bursting broadly follow a time course similar to that of the waveform of the average EPSP generated in response to HT bursting network activity, except that the peak in firing is slightly delayed compared to the peak of the average EPSP (time from HT burst start to peak in interneuron firing: 56.4 ± 7.4 ms; n = 3) (Figure 7D, top and gray bars on right). In contrast, when repetitive bursting is absent, interneuron firing is most prominent at the point of average EPSP onset (4.3 ± 0.7 ms; p > 0.1; n = 6) (Figure 7D, bottom and black bars on right).

## Discussion

**Figure 8:**
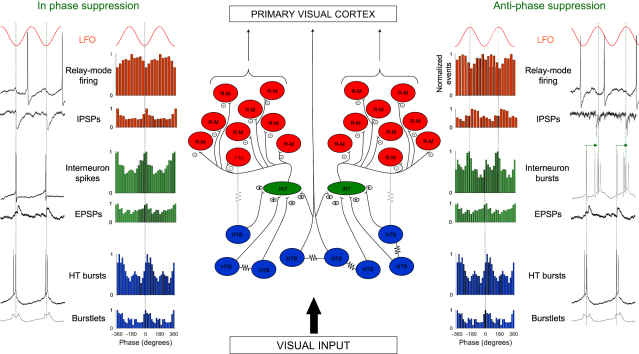
Schematic Representation of the Thalamic Circuitry Underlying the α-Rhythm-Derived Temporal Framing of Relay-Mode Firing HT bursting TC neurons (blue filled circles, HTB) form a GJ-coupled network in the LGN that generates synchronized oscillations at α frequencies. These cells may also couple via weak GJs (thin dotted lines) to a subset of TC neurons that exhibit conventional relay-mode firing (red filled circles, R-M). The main sequence of α-rhythm-related events that shape relay-mode firing, however, is as follows. First, HT bursting TC neurons provide a convergent excitation of local GABAergic interneurons (green filled circles, INT), probably via axon collaterals. This convergent excitation rhythmically drives interneuron firing, which can consist of either primarily single spike output (left side) or bursting (right), with bursting occurring when interneurons occupy a more excited state (see Figure 6B). During single spike output, action potentials in interneurons occur predominantly close to the negative α rhythm peak. In contrast, during bursting, a large interval between the first and second spikes in individual bursts means that action potentials are translated to occur mainly close to the positive α rhythm peak. These two forms of interneuron firing are then reflected in relay-mode TC neurons as mainly single IPSPs that occur near the negative α rhythm peak or IPSP bursts that occur near the positive α rhythm peak, respectively. Ultimately, these two forms of inhibition lead to a differential temporal framing of output from relay-mode TC neurons. Since HT bursting neurons and relay-mode cells are both TC neurons, they both receive retinal input, and send projections to the primary visual cortex. This means (1) that the α-rhythm-generating machinery in the LGN can be potentially perturbed or entrained by sensory input, and (2) that the neocortex not only receives modulated input from relay-mode TC neurons, but also receives a signal from the rhythmic network of cells that provides that modulation.

The main finding of this study is that during the α rhythm, LGN relay-mode TC neurons form two distinct coalitions, one of which shows a cyclic suppression of firing that is centered on the negative peak of the α rhythm, and the other of which displays a cyclic suppression centered on the positive peak. This leads to an effective temporal framing of action potential output relative to the ongoing rhythm. Membership of these two alliances is simply determined by whether cells receive interneuron-derived phasic inhibition predominantly close to the negative or positive α rhythm peak, with the latter form of inhibition representing input from interneurons that occupy a more excited state (Figure 8). These results provide a potential cellular substrate for linking the α rhythm to perception and further emphasize the critical role of inhibition in controlling spike timing during cognitively relevant brain rhythms (Klausberger and Somogyi, 2008).

The mechanistic insights obtained in this study are largely provided by extensive in vitro experiments on a thalamic slice preparation that retains the capacity to generate network α oscillations (Lőrincz et al., 2008). This approach is justified by several points of striking correspondence between our in vivo and in vitro findings. Importantly, these aspects of similarity are only present in the slice preparation following cholinergic receptor activation and are not mimicked following mGluR activation. This is consistent with our in vivo microdialysis experiments that indicate that LGN α activity and related action potential firing are mainly supported by a cholinergic drive, with a seemingly lesser role for mGluRs. Indeed, we find that while mGluR activation in vitro may well bring about synchronized HT bursting (Hughes et al., 2004), it also leads to unrestrained firing in PGN neurons, which in turn causes indiscriminate inhibition and uncoordinated firing in relay-mode TC neurons.

Our results demonstrate unequivocally that the temporal framing of relay-mode TC neuron activity results from phasic inhibition from LGN interneurons. However, a crucial component in ultimately bringing about this temporal framing is the rhythmic synaptic excitation of interneurons, because without this excitation these cells do not generate action potentials and the phasic inhibition of relay-mode TC neurons does not occur. Given that this excitation occurs at a similar frequency to HT bursting and that individual EPSPs correlate with both multiunit and single-unit HT bursting activity, we can only reasonably conclude that LGN interneurons receive a convergent excitation from the HT bursting TC neuron network (Lőrincz et al., 2008), with this most likely occurring via intranuclear axon collaterals (Cox et al., 2003; Bickford et al., 2008). Interneurons may well also receive direct connections from relay-mode TC neurons, but since firing in these cells was never found to be correlated with synaptic events in interneurons, this pathway appears, at the very least, to be overwhelmed by the powerful postsynaptic effect of synchronized HT bursting as one would intuitively expect. On the other hand, the general lack of correlation between excitatory synaptic input to PGN neurons (which could at times be quite prominent) and LGN α activity suggests that there might be some specificity in the connections from HT bursting TC neurons to interneurons that is not shared by the TC neuron-PGN neuron innervation. With regard to why mGluR activation does not bring about a similar rhythmic excitation of inteneurons despite leading to synchronized HT bursting in subsets of TC neurons (Hughes et al., 2004), we suspect that, following mGluR activation, such synchronization occurs to a much lesser extent than during cholinergic activation, as indicated by the substantially larger-amplitude α frequency field oscillations that are observed in vitro following Cch application (∼100–200 μV versus ∼40–50 μV) (Hughes et al., 2004; Lőrincz et al., 2008), leading in turn to a less coordinated overall output.

The finding that LGN interneurons can generate action potentials in both a single spike and burst mode is consistent with previous in vitro studies that have shown that bursting in LGN interneurons (1) is an intrinsic phenomenon that is primarily reliant on Ca^2+^ channels (Zhu et al., 1999a, 1999b; Pape et al., 1994; Acuna-Goycolea et al., 2008), (2) can occur rhythmically at relatively depolarized membrane potentials (Zhu et al., 1999a, 1999b; Pape et al., 1994; Cox et al., 2003; Acuna-Goycolea et al., 2008), (3) can be occluded in normal conditions by outward K^+^ conductances, hinting that changes in neuromodulatory tone might readily alter the propensity to burst (Pape et al., 1994), and (4) can be engaged by rhythmic (Cox et al., 2003) or isolated (Zhu et al., 1999a; Acuna-Goycolea et al., 2008) EPSPs, again from relatively depolarized membrane potentials. Of particular relevance here is the recent observation by Acuna-Goycolea et al. (2008) that action potential bursts in mouse LGN interneurons can lead to bursts of IPSPs in neighboring relay cells that are similar to those shown here.

Phasic inhibition onto TC neurons is also a key component in generating thalamic spindle oscillations (Andersen and Andersson, 1968; von Krosigk et al., 1993). However, fundamental differences exist with the scenario described here. Most notably, phasic inhibition during α activity is derived from probably one or very few LGN interneurons, leading to subtle IPSPs (∼0.5–1 mV) that delicately control action potential output, whereas inhibition during spindles originates from widespread bursting in the PGN, leading to large-amplitude IPSPs (∼10–15 mV) in TC neurons (von Krosigk et al., 1993). Also, during α rhythms relay-mode TC neurons that are subject to phasic inhibition are relatively depolarized and generate action potentials solely in a relay-mode, while during spindles TC neurons are hyperpolarized and produce action potentials in low-threshold bursts (von Krosigk et al., 1993). These differences are clearly commensurate with the fact that spindle waves are pervasive brain oscillations that occur during sleep and anesthesia, whereas α activity is a more localized, responsive rhythm of the waking brain (Hughes and Crunelli, 2005).

Despite the definitive status of the α rhythm as a wake-related EEG hallmark, its preeminence in the eyes closed condition and its classical suppression upon eye opening have led to a common belief that it represents an idling of visual cortical areas. However, a long history of experimental observations from humans not only shows that the α rhythm also occurs abundantly when the eyes are open, but also that it is closely linked to the timing of certain types of perceptual events (Bartley, 1940; Lindsley, 1952; Lansing, 1957; Anliker, 1963, 1966; Mathewson et al., 2009) such as reaction time (Surwillo, 1961) and perceived simultaneity (Kristofferson, 1967). In conjunction with various other key psychophysiological findings, this has led to the suggestion that the α rhythm supplies a timing signal for discrete, periodic perceptual sampling, particularly within the visual system (Crick and Koch, 2003; VanRullen and Koch, 2003; Fingelkurts and Fingelkurts, 2006; Mathewson et al., 2009), thus ascribing it a role similar to that of oscillations in other sensory systems, such as rodent olfaction and somatosensation (i.e., active whisking) (Uchida et al., 2006). Our study provides potential cellular-level support for this idea because it demonstrates that naturally occurring α rhythms actively and discretely constrain the temporal dynamics of neurons that directly carry out the transmission, and influence the processing, of early-stage visual information. Indeed, a cyclic facilitation of relay-mode firing in LGN TC neurons for ∼70%–80% of the α rhythm cycle (Figure 8) closely matches the typical estimates of the duration of a discrete processing epoch in human perception (Stroud, 1955; Efron, 1970; VanRullen and Koch, 2003; Fingelkurts and Fingelkurts, 2006).

## Experimental Procedures

All in vivo and in vitro experiments were carried out in accordance with the guidelines of the local ethical committees, the UK Animals (Scientific Procedure) Act, 1986 and the Hungarian Act of Animal Care and Experimentation (1998, XXVIII, Section 243/1998), which conforms to the European Community regulations (86/609/). All efforts were made to minimize the suffering and number of animals used in each experiment.

### Surgery and Implantation for In Vivo Recordings

Surgery for chronic implantation was carried out as described previously (Hughes et al., 2004). Briefly, adult cats (3.2–4.5 kg) were anaesthetized with 40 mg/kg Nembutal and placed in a stereotaxic frame (David Kopf 900 series, David Kopf Instruments, Tujunga, USA). Stainless steel screws (0.8 mm) were implanted above the occipital and parietal cortices for EEG recording. Bilateral 3 mm holes were drilled into the bone for implanting electrode arrays (see below) at coordinates A: 7.2, L: 9.5–10, V: +6 mm (Berman and Jones, 1982). These are located in lamina A of the LGN and correspond to an area that we have previously identified as being important for α rhythm generation (Hughes et al., 2004; Hughes and Crunelli, 2005; Lőrincz et al., 2008). Cats were allowed to recover from the implantation for at least 5 days before recording commenced. For recording extracellular unit activity and LFPs from the LGN, cats were chronically implanted with microelectrodes. Two custom-made bundles consisting of 8 or 16 Teflon-insulted 25 μm Pt/Ir wires and 1 80 μm Ni/Cr wire (150–300 kΩ impedance at 1 kHz) were attached to the outer walls of a 22G polyethylene guide cannula such that the tips of the electrodes were 10 mm below the base of the cannula. The guide cannula was then attached to the piston of a cylinder-piston-type microdrive (Hughes et al., 2004). Local thalamic drug application was enabled using reverse microdialysis. Custom-made microdialysis probes (4 mm length, hollow fiber, 50 kDa molecular weight cut-off, 200 μm outer diameter) were inserted into 27G stainless steel tubing. This stainless steel tubing was then inserted into the polyethylene guide cannula. Once inserted, the microdialysis probe was fixed with two screws to the top of the metal cylinder to prevent any further movement. The turning of the piston thread, therefore, only caused the electrode bundles to slide, while the probe was maintained in a constant position. Additional information relating to the acquisition and analysis of in vivo electrophysiological data is given in the Supplemental Data.

### In Vitro Slice Preparation and Maintenance and In Vitro Electrophysiology

Young adult cats (1–1.5 kg) were deeply anaesthetized with a mixture of O_2_ and NO_2_ (2:1) and 2.5% halothane, a wide craniotomy was performed, and the brain was removed. Sagittal slices of the LGN-PGN were prepared and maintained as described previously (Hughes et al., 2002, 2004; Lőrincz et al., 2008). For recording, slices (450–500 μm) were perfused with a warmed (35°C ± 1°C), continuously oxygenated (95% O_2_, 5% CO_2_) artificial cerebrospinal fluid (ACSF) containing (in mM) NaCl (134), KCl (2), KH_2_PO_4_ (1.25), MgSO_4_ (1), CaCl_2_ (2), NaHCO_3_ (16), and glucose (10). Extracellular single-unit and field recordings were performed using glass pipettes filled with 0.5 M NaCl (resistance: 1–5 MΩ) connected to a Neurolog 104 differential amplifier (Digitimer Ltd., Welwyn Garden City, UK). Independently mounted intracellular recordings, using the current-clamp technique, were performed with standard-wall glass microelectrodes filled with 1M potassium acetate (KAc) or 2M ceasium acetate and 50 mM QX314 (resistance: 80–120 MΩ), and in some cases 2% biocytin or neurobiotin, and connected to an Axoclamp-2A amplifier (Axon Instruments, Foster City, CA, USA) operating in bridge mode. Additional information relating to the acquisition and analysis of in vitro electrophysiological data is given in the Supplemental Data.

## Supplemental Data

Document S1. Supplemental Results, Experimental Procedures, Figures, and Tables
