# Supplementary material for: Temporal Framing of Thalamic Relay-Mode Firing by Phasic Inhibition during the Alpha Rhythm
Source: Neuron. 2009 Sep 10;63(5):683–96. doi: 10.1016/j.neuron.2009.08.012 (PMC2791173; doi:10.1016/j.neuron.2009.08.012)
Supplement: Document S1. Supplemental Results, Experimental Procedures, Figures, and Tables [file mmc1.pdf]

Neuron, volume 63
Supplemental Data

Temporal Framing of Thalamic Relay-Mode Firing by Phasic Inhibition during the Alpha Rhythm

Magor L. Lőrincz, Katalin A. Kékesi, Gábor Juhász, Vincenzo Crunelli, and Stuart W. Hughes

Supplementary results

A. Local pharmacological inhibition of mAChRs or mGluR1a in the LGN *in vivo* suppresses HT bursting, relay mode firing and associated α activity

Whilst both HT bursting and tonic/relay-mode firing occur when TC neurons are relatively depolarized, *in vitro* experiments in isolated LGN slices have shown that HT bursting, and associated field oscillations in the α band, arise as a specific response to the depolarization brought about by activating either muscarinic acetylcholine receptors (mAChRs) (Lőrincz et al., 2008, 2009) and/or metabotropic glutamate receptor 1a (mGluR1a) (Hughes et al., 2004; Lőrincz et al., 2009). This suggests that in intact animals the expression of HT bursting and α activity in the LGN may also require the specific activation of these receptors. In order to investigate more directly to what extent activation of mAChRs and mGluR1a support HT bursting and α rhythms in the LGN during natural wakefulness we applied either the mAChR antagonist pirenzipine (0.1-1 mM, in the probe) or the mGluR1a antagonist, LY367385 (0.5-4 mM, in the probe) directly to the LGN of naturally waking cats via reverse microdialysis, whilst monitoring EEG, local field oscillations (LFOs) and local unit firing. Both agents caused a reversible and dose-dependent decrease in both the prevalence of HT bursting (Supp. Fig. 5A and Supp. Table 1) and the mean rate of tonic/relay-mode firing (Supp. Fig. 5B and Supp. Table 1). Furthermore, these effects were accompanied by a dose-dependent reduction of both LGN and EEG α activity (Supp. Table 2). Notably, the maximum reduction in HT bursting and LGN α activity brought about by pirenzipine was significantly greater than that brought about by LY367385 ($p<0.01$) (Supp. Fig. 5 and Supp. Tables 1 and 2). These results therefore suggest that the presence of HT bursting and α waves in the LGN is primarily, though not exclusively, due to mAchR activation. This is consistent with slice experiments where activation of mAchRs leads to substantially larger α rhythms (~100-200 μV) (Lőrincz et al., 2008) than activation of mGluR1a (~40-50 μV) (Hughes et al., 2004). Importantly, this data also indicates that action potential output from LGN TC neurons is a primary influence on EEG α rhythm generation.

B. Are relay-mode TC neurons always suppressed at the same phase?

Within the timescale of our unit recordings *in vivo*, relay-mode TC neurons were on average clearly inhibited either close to the negative or positive peak of the local field oscillation. That said, closer scrutiny showed that even cells that were, for example, mainly inhibited near one peak could also show brief periods where they were suppressed near the other peak but in a manner which was not statistically significant (due to a small sample of spikes for that brief period). This is clearly consistent with the variable interneuron activity described for both *in vivo* and *in vitro* recordings (see Figs. 6 and 7 and Supp. Figs. 10 and 11) and suggests that ultimately the local level of excitability, which will certainly vary considerably over an extended time-course, may lead to dynamic changes in the phase at which the suppression of firing occurs in relay-mode TC neurons.

C. Evidence for inconsistent and weak gap junction (GJ)-mediated connections between HT bursting and relay-mode TC neurons

In some relay-mode TC neurons (n=5) recorded in normal conditions *in vitro*, in addition to IPSPs we sometimes observed low-amplitude, fast depolarizing events (duration: 16.3±2.3 ms; amplitude: 0.9±0.1; n=20 events) (Supp. Fig. 7A; see also blue asterisks in Supp. Fig. 6A and 6E). These events were similar to spikelets observed in our previous studies (Hughes et al., 2002a, 2004; Lőrincz et al., 2008, 2009; see especially Fig. 9 in Lőrincz et al., 2008), were slightly more common following GBZ application (control events/s: 0.04±0.03; GBZ events/s: 0.14±0.07; n=3) (Supp. Fig. 6E) and probably represent a weak and inconsistent GJ-mediated connection of relay-mode TC neurons to the HT bursting TC neuron network (Lőrincz et al., 2008). Indeed, in two TC neurons recorded using Cs^+- and QX314-filled electrodes, application of GBZ not only abolished rhythmic IPSPs but also unmasked clear burstlets (i.e. HT bursts that have been communicated through an electrical synapse; see Hughes et al., 2004, Lőrincz et al., 2008, 2009) (peak amplitude: 1.1±0.1 mV; n=2) (Supp. Fig. 7B) that were similar, although smaller in amplitude, to those observed in HT bursting TC neurons (see below) and which occurred at a comparable frequency (HT bursting frequency: 7.61±0.8 Hz; burstlet frequency: 8.1±0.5 Hz; n=13 and 2) (Supp. Fig. 8A). Interestingly, and in relation to this, following a disruption of temporal framing in relay-mode TC neurons by GBZ, overall firing in both the in-phase (Fig. 5A, right) and anti-phase (Fig. 5B, right) suppressed groups became characterised by a spike timing histogram that exhibited two small transient elevations close to the negative and positive α rhythm peaks. Although these firing distributions did not exhibit significant non-uniformity (i.e. rhythmicity) in either case, it is nevertheless tempting to speculate that they may result from a weak, residual GJ-mediated attachment to the HT bursting TC neuron population.

D. HT bursting TC neurons recorded in the presence of Cch *in vitro* do not generally appear to be the recipients of phasic inhibition

In 3 of the 5 TC neurons that did not exhibit IPSPs during recordings with Cs^+- and QX314-filled electrodes we observed rhythmic burstlets (peak amplitude: 3.6±1.0 mV; n=3) that were tightly correlated with the negative LFO peak ($p<0.05$; Rao's test; n=3) and which, again, occurred at an equivalent frequency to HT bursting (burstlet frequency: 7.3±0.7 Hz; $p>0.1$; n=3) (Supp. Fig. 8A, top). Given the lack of overt IPSPs but clear presence of burstlets in normally-recorded HT bursting TC neurons (Lőrincz et al., 2008, 2009) (Supp. Fig. 8A, bottom), it is likely that these cells represent HT bursting TC neurons. Unfortunately, the presence of Cs^+ and QX314 in the electrode rapidly altered firing properties to such an extent that we were unable to ascertain this directly. However, if these cells do indeed represent HT bursting TC neurons, then the lack of IPSPs predicts that blocking fast GABAergic synaptic transmission should have no effect on the properties of HT bursting when assessed with normal recordings. Indeed, application of GBZ to conventionally-recorded (i.e. with KA-filled electrodes) HT bursting TC neurons did not affect either bursting properties (Supp. Fig. 8B) or the association between bursting and the LFO (Supp. Fig. 8B). In fact, whilst HT bursting TC neurons often exhibited IPSPs in control conditions, IPSPs were actually abolished in all (n=10) but one HT bursting TC neuron by the addition of Cch (Supp. Fig. 8C). These findings suggest either that HT bursting TC neurons do not receive direct connections from LGN interneurons or that they receive input from a population of interneurons which are quiescent following Cch application and which have gone undetected in this study. Were the latter to be true, one possibility is that different LGN interneurons may respond differentially to activation of Ach receptors, perhaps showing either a mainly muscarinic or nicotinic response, as is the case for some types of cortical interneurons (Xiang et al., 1998)

E. Bursting in LGN interneurons is an intrinsic phenomenon

As indicated by several earlier studies (Zhu et al., 1999a,b; Pape et al., 1994; Cox. et al., 2003; Acuna-Goycolea et al., 2008), burst firing was found to be an intrinsic capability of LGN interneurons because bursts could also be elicited at the offset of injected negative current steps and were resistant to blocking fast excitatory synaptic transmission with a combination of 20 μM NBQX and 50 μM APV (n=8) (Supp. Fig. 11B and D). Interestingly, in interneurons that did not burst in the presence of Cch, combined NBQX/APV application unmasked this activity (Supp. Fig. 11B) suggesting that the increase in membrane conductance brought about by intense synaptic activity can sometimes prevent bursting (see Zhu et al., 1999a; Pape et al., 1994). In all cases, bursts in interneurons elicited by injection of current pulses were of high frequency (mean ISI: 5.8±0.5 ms; n=8) and showed a clear accelerando-decelerando pattern, similar to that shown previously for neurons from the TRN (Domich et al., 1986; Bal and McCormick, 1993).

F. Relay-mode TC neurons show an inhibition and suppression of firing coincident with action potential output from interneurons

To strengthen the case that inhibition in relay-mode TC neurons is related to the activity of LGN interneurons, we performed intracellular recordings from relay-mode TC neurons *in vitro* whilst monitoring the output of closely situated (<200 μm) interneurons. These often showed that action potential output from interneurons is, when relay-mode TC neuron activity is averaged over several oscillation cycles, temporally related to IPSP generation in these cells (time from interneuron burst start to average IPSP onset: 5.2±1.0 ms; amplitude: 0.4±0.07 mV; n=7 pairs) (Supp. Fig. 12A) In some cases (n=2 of 7), these IPSPs were accompanied by bursts of small spikelets (i.e. action potentials from HT burst TC neurons transmitted via a weak electrical synapse, see above) (Supp. Fig. 12A; see also above, Supp. Fig. 6A and E and Supp. Fig. 7) (Hughes et al., 2002a, 2004; Lőrincz et al., 2008, 2009), thus allowing a simultaneous appreciation of the full sequence of network events, from firing in HT bursting TC neurons and interneurons to the ultimate shaping of activity in relay mode TC neurons. Interestingly, mirroring the relationship between interneurons and relay-mode TC neurons *in vivo* (Fig. 3D), double unit extracellular recordings of a relay-mode TC neuron and a closely situated interneuron revealed in all cases that interneuron activity was coincident with a robust suppression of relay-mode firing (78.3±9.2%; n=3 pairs) (Supp. Fig. 12B). HT bursting TC neurons, on the other hand did not exhibit any form of inhibition coincident with neighbouring interneuron firing (data not illustrated).

G. Evidence that LGN interneurons receive excitatory input from HT bursting TC neurons

In some LGN interneurons recorded *in vitro* during Cch application we were able to identify rhythmic EPSP complexes which clearly mirrored the output of HT bursting TC neurons (1-3 EPSPs per complex; inter-EPSP interval range: ~10-20 ms) (Supp. Fig. 13B, top). Interestingly, during Cch washout, these complexes initially ceased before a different type of EPSP complex later appeared at a lower frequency (~0.5-2 Hz) (Supp. Fig. 13B, bottom). In particular, the timing of individual EPSPs in these distinct complexes showed an initial higher frequency of individual EPSPs (inter-EPSP interval range: ~3-6 ms) and a lengthening of the inter-EPSP interval as the complex progressed (Supp. Fig. 13B, bottom). This pattern of synaptic input matches the action potential timing observed during LTCP-mediated bursts in TC neurons (see extracellular recording in Supp. Fig. 13B, bottom) (see also Fig. 3 in Lőrincz et al. 2008). Indeed, the initial loss of HT burst-matching EPSP complexes and then their replacement in well-defined bursts at a lower frequency precisely mirrors that

which occurs to action potential output in HT bursting TC neurons during washout of Cch (see extracellular recordings in Supp. Fig. 13B) and confirms that LGN interneurons receive excitatory synaptic input from TC neurons (Cox et al., 2003; Bickford et al., 2008).

H. Relay mode TC neurons do not show temporal framing following pharmacological activation of mGluRs due to indiscriminate inhibition in TC neurons

In contrast to that observed in the presence of Cch, but consistent with the finding that LGN α rhythms recorded during natural wakefulness in intact animals appear to be primarily supported by a cholinergic drive (Supp. results section A, Supp. Tables 1 and 2 and Supp. Fig. 5), none out of 20 relay-mode TC neurons recorded in LGN slices during α rhythms that had been brought about by the mGluR agonist, *trans*-ACPD (100 μM) (see Hughes et al., 2004), showed significant correlations with the ongoing LFO (p>0.1; Rao's test) (Supp. Fig. 14A). This was because in the presence of 100 μM *trans*-ACPD, although normally-recorded relay-mode TC neurons often displayed occasional discrete IPSPs (data not illustrated), when recorded using Cs^+/QX314-filled electrodes, TC neurons exhibited barrages of IPSPs that were non-rhythmic and that occurred at a significantly higher mean frequency than during Cch application (*trans*-ACPD: 54.4±5.4 events/s; Cch: 11.5±2.4 events/s; n=15 and 16; p<0.001) (Supp. Fig. 14B).

Intracellular recordings of LGN interneurons and PGN neurons obtained in the presence 100 μM *trans*-ACPD revealed that indiscriminate inhibition was likely present in relay-mode TC neurons because, whilst interneurons exhibited only a slightly higher rate of firing compared to that observed during Cch application (5.8±0.8 *vs* 3.3±0.2spikes/s; n=15 and 20; p>0.1) (Supp. Fig. 15A1, top trace, and 15C) and that sometimes consisted of rhythmic bursting (Supp. Fig. 15A2, top trace), PGN neurons displayed indiscriminate action potential output at a considerably higher frequency (34.5±2.4 *vs* 0.03±0.005 spikes/s; n=15 and 11; p<0.001) (Supp. Fig. 15B1 and 15C, left) (see also Blethyn et al., 2006). Also, in the presence of *trans*-ACPD, whilst both cell types received ongoing barrages of excitatory synaptic activity (Supp. Fig. 15A1 and A2, bottom traces, and 15B2), reflecting input from an active TC neuron population, these EPSP complexes were essentially non-rhythmic and displayed an amplitude that for LGN interneurons was smaller compared to that observed in the presence of Cch (2.2±0.4 *vs* 5.1±0.5 mV; n=13 and 20; p<0.001) but for PGN neurons was comparable (2.8±0.5 *vs* 2.3±0.4 mV; n=11 for both cases; p>0.1) (Supp. Fig. 15A and B, auto-correlograms on right, and Supp. Fig. 15C, middle). Finally, individual EPSPs in both LGN interneurons occurred much less frequently following *trans*-ACPD application than following Cch treatment (5.0±0.8 *vs* 18.8±2.6 event/s; n=14 and 20; p<0.001), whereas in PGN neurons they were more common (9.6±2.2 *vs* 3.8±1.1 events/s; n=15 and 11; p>0.1) (Supp. Fig. 15C, right).

Supplementary methods

In vivo recording and behavioural protocol

During the experiments the animals were allowed to move freely in a sound-proofed and electrically-shielded chamber (150 x 100 x 170 cm). Stages of vigilance were determined from a combination of EEG and behavioural criteria as fully described previously (Hughes et al., 2004). During episodes of α activity cats were in a state of quiet or relaxed wakefulness and generally assumed the 'sphinx' position. As such, occurrences of α waves did not correspond to any overt behavior and we did not set out to correlate α activity with any specific behavioral task or context. Rather, periods of activity were chosen according to the amount of α activity exhibited in the LFP, specifically defined as periods when α waves were at least twice the amplitude of normal baseline activity (Hughes et al., 2004). Accordingly, α rhythm density was defined as the percentage of time taken up by rhythmic α activity defined in such a way. Extracellular signals were amplified by a Supertech Multiamp amplifier (Supertech, Pécs, Hungary) and then digitally acquired with a CED 1401 (Cambridge Electronic Design Ltd., Cambridge, UK) for storage and offline analysis on a personal computer or were amplified and acquired by a 64 channel Plexon integrated recording system (Plexon Inc., Dallas, USA). Unless explicitly stated otherwise, field and unit signals were isolated by post-hoc digital band-pass filtering at 2-20 Hz and 0.3-10 kHz, respectively. At the end of an experiment, animals were given a lethal injection of Nembutal.

In vivo data analysis and neuron classification

Recorded units were identified as TC neurons on the basis that during deep sleep they exhibited unequivocal low-threshold Ca^{2+}-potential (LTCP)-mediated bursts (Domich et al., 1986; Steriade et al. 1993; Hughes et al. 2004). An LTCP-mediated burst was defined as a group of two or more spikes exhibiting a first ISI of $\leq$6 ms, subsequent intervals of <15 ms which progressively increased, and a preceding silent period of at least 200 ms. HT bursts were defined as a group of two or more spikes exhibiting a preceding silent period of at least 70 ms and ISIs that were between 3 and 20 ms and which (for bursts of more than 2 spikes) did not systematically increase as the burst progressed. All TC neurons that did not exhibit HT bursts were classified as tonic firing/relay-mode neurons. The definitions of both HT and LTCP-mediated bursts were based on extensive intracellular recordings from the cat LGN (Pirchio et al., 1997; Hughes et al., 2002b, 2004; Lőrincz et al., 2008). As described above to be classified as originating from TC neurons, single unit recordings from the LGN of freely moving cats were required to exhibit unequivocal LTCP mediated bursts during periods of deep sleep (Domich et al., 1986; Steriade et al. 1993; Hughes et al. 2004). Interestingly, in the course of our investigations we recorded several LGN cells which did not fulfil the criteria to be classified as TC neurons (given above), that showed a distinct extracellular action potential waveform (Supp. Fig. 3A), and which we therefore assumed to be local circuit interneurons (Pape and McCormick, 1995; Williams et al., 1996). PGN neurons were identified using stereotaxic coordinates, electrode tracks and by the presence of characteristic prolonged bursts of action potentials during deep sleep (Domich et al, 1986). For all animals, electrode tracks and final tip positions were confirmed post mortem in histological sections, providing further verification that recordings had been obtained from the presumed sites in either in the LGN or PGN.

Phase values of neuronal firing were analyzed by circular statistical methods using Oriana 2.0 software (Kovach Computing Services, Anglesey, UK). Significant non-uniformity in the phase of firing relative to the ongoing LFO was tested for with Rao's spacing test ($p<0.05$). This test calculates the probability of the null hypothesis that

the data are distributed in a uniform manner. Comparison of circular distributions was performed with the Mardia-Watson-Wheeler test and the Watson U^2 test. Both are non-parametric tests where the null hypothesis is that the two distributions under examination are identical. Absolute spike times were determined using a straightforward visually-determined threshold detection approach. To construct a spike timing histogram, the times of at least 200 spikes (comprising groups of consecutive spikes from several consecutive α rhythm epochs) were determined relative to the nearest negative peaks of the LFO using custom written transform routines in SigmaPlot 9 (Systat, Hounslow, UK). These times were subsequently assigned a given phase between these peaks (i.e. between 0° and 360°) and then binned at 24°-36°. For clarity, and to afford a sense of rhythmicity, this data was usually repeated over at least one additional full cycle of the oscillation (-360 ^{o} to 0°) to produce the final plot (Klausberger et al., 2003,2004; Hájos et al., 2004; Lőrincz et al., 2008; Hughes et al. 2008). Cross-correlograms of simultaneously recorded units were constructed from 10 consecutive α rhythm epochs. Cell pairs were considered to be correlated if the corresponding cross-correlogram exhibted a central peak or trough which exceeded the 95% confidence limit. Unless otherwise stated all quantitative *in vivo* data are expressed as mean±s.e.m and statitistical significance was assessed with either Student's t-test or an ANOVA test.

***In vitro* data analysis and neuron identification**
Impaled cells were identified as TC neurons using established criteria (Hughes et al., 2002a,b, 2004; Lőrincz et al., 2008). Interneurons were identified morphologically using established criteria (McCormick and Pape, 1988; Pape and McCormick, 1995; Williams et al., 1996; Cox et al., 2003) and electrophysiologically by a significantly shorter action potential half width and smaller action potential afterhyperpolarization (see Supp. Fig. 3) (McCormick and Pape, 1988; Pape and McCormick, 1995; Williams et al., 1996). Definitive recordings of PGN neurons were obtained as described previously (Blethyn et al. 2006). Voltage and current records were digitally acquired and processed using pClamp 9 (Molecular Devices Corporation, Sunnyvale, CA, USA).

Phase values for neuronal firing and significant non-uniformity in neuronal firing relative to the ongoing LFO or extracellular rhythmic bursting were assessed as *in vivo*. Spike, IPSP and EPSP timing histograms were constructed as for *in vivo* data except that the absolute timing of individual events was calculated from their positive or negative peak. Grand averages of LFO-triggered average subthreshold membrane potential traces was produced after first time normalizing the data between either consecutive negative peaks for in-phase suppressed cells (i.e. 0 and 360°) or consecutive positive peaks for anti-phase suppressed cells (i.e. -180 and $+180^o$). Auto- and cross-correlation functions were computed using using custom written transform routines in SigmaPlot 9. Trains of IPSPs or EPSPs were considered to be rhythmic if their corresponding auto-correlograms exhibited clear satellite peaks at consistent intervals. As with *in vivo* data, unless otherwise stated all quantitative *in vitro* data are expressed as mean±s.e.m. and statitistical significance was assessed with Student's t-test

Sources of drugs:
DL-2-amino-5-phosphonovaleric acid (APV), (S)-(+)-a-amino-4-carboxy-2-methylbenzeneacetic acid (LY367385), 2,3-dioxo-6-nitro-1,2,3,4-tetrahydrobenzo[f]quinoxaline-7-sulfonamide disodium salt (NBQX), N-(2,6-dimethylphenylcarbamoylmethyl) triethylammomium bromide (QX314), 6-imino-3-(4-methoxyphenyl)-1(6*H*)-pyridazinebutanoic acid hydrobromide (SR95531, GBZ) from Tocris-Cookson (UK); carbamylcholine chloride (carbachol, Cch), 5,11-dihydro-11-

([4-methyl-piperazin]acetyl)-6H-pyriso(2,3-b)(1,4)benzodiazepine-6-one (pirenzipine, Pzp) were obtained from Sigma (UK). All drugs were dissolved in ACSF.

Supplementary references

Acuna-Goycolea, C., Brenovitz, S.D., and Regehr, W.G. (2008). Active dendritic conductances dynamically regulate GABA release from thalamic interneurons. Neuron *57*, 420-431.

Bal, T, and McCormick, D.A. (1993). Mechanisms of oscillatory activity in guinea-pig nucleus reticularis thalami in vitro: a mammalian pacemaker. J. Physiol. (London) *468*, 669-691.

Bickford, M.E., Wei, H., Eisenback, M.A., Chomsung, R.D., Slusarczyk, A.S., and Dankowsi, A.B. (2008). Synaptic organization of thalamocortical axon collaterals in the perigeniculate nucleus and dorsal lateral geniculate nucleus. J. Comp. Neurol. *508*, 264-285.

Blethyn, K.L., Hughes, S.W., Tóth, T.I., Cope, D.W., and Crunelli V. (2006). Neuronal basis of the slow (<1 Hz) oscillation in neurons of the nucleus reticularis thalami in vitro. J. Neurosci. 26, 2474-2486.

Cox, C.L., Reichova, I, and Sherman, S.M. (2003). Functional synaptic contacts by intranuclear axon collaterals of thalamic relay neurons. J. Neurosci. *23*, 7642-7646.

Domich, L., Oakson, G., and Steriade, M. (1986). Thalamic burst patterns in the naturally sleeping cat: a comparison between cortically projecting and reticularis neurones. J. Physiol. (London) *379*, 429-449.

Hájos, N., Pálhalmi, J., Mann, E.O., Németh, B., Paulsen, O., and Freund, T.F. (2004). Spike timing of distinct types of GABAergic interneuron during hippocampal gamma oscillations in vitro. J. Neurosci. *24*, 9127-9137.

Hughes, S.W., Blethyn, K.L., Cope, D.W., and Crunelli, V. (2002a). Properties and origin of spikelets in thalamocortical neurones in vitro. Neuroscience *110*, 395-401.

Hughes, S.W., Cope, D.W., Blethyn, K.L., and Crunelli, V. (2002b). Cellular mechanisms of the slow (<1 Hz) oscillation in thalamocortical neurons in vitro. Neuron *33*, 947-958.

Hughes, S.W., Lörincz, M., Cope, D.W., Blethyn, K.L., Kékesi, K.A., Parri, H.R., Juhász, G., and Crunelli, V. (2004). Synchronized oscillations at alpha and theta frequencies in the lateral geniculate nucleus. Neuron *42*, 253-268.

Hughes, S.W., Lörincz, M., Cope, D.W., and Crunelli, V. (2008). NeuReal: an interactive simulation system for implementing artificial dendrites and large hybrid networks. J. Neurosci. Methods *169*, 290-301.

Klausberger, T., Magill, P.J., Márton, L.F., Roberts, J.D., Cobden, P.M., Buzsáki, G., and Somogyi, P. (2003). Brain-state- and cell-type-specific firing of hippocampal interneurons in vivo. Nature *421*, 844-848.

Klausberger, T., Márton, L.F., Baude, A., Roberts, J.D., Magill, P.J., and Somogyi, P. (2004). Spike timing of dendrite-targeting bistratified cells during hippocampal network oscillations in vivo. Nat. Neurosci. *7*, 41-47.

Lőrincz, M.L., Crunelli, V., and Hughes, S.W. (2008). Cellular dynamics of cholinergically-induced alpha (8-13 Hz) rhythms in sensory thalamic nuclei in vitro. J. Neurosci. *28*, 660-671.

Lőrincz, M.L., Geall, F., Bao, Y., Crunelli, V., and Hughes, S.W. (2009). ATP-dependent infra-slow (<0.1 Hz) oscillations in thalamic networks. PLoS ONE *4*, e4447.

McCormick, D.A., and Pape, H.C. (1988). Acetylcholine inhibits identified interneurons in the cat lateral geniculate nucleus. Nature *334*, 246-248.

Pape, H.-C., Budde, T., Mager, R., and Kisvárday, Z.F. (1994). Prevention of Ca(2+)-mediated action potentials in GABergic local circuit neurones of rat thalamus by transient K^+ current. J. Physiol. (Lond). *478*, 302-422.

Pape, H.-C., and McCormick, D.A. (1995). Electrophysiological and pharmacological properties of interneurons in the cat dorsal lateral geniculate nucleus. Neuroscience *68*, 1105-1125.

Pirchio, M., Turner, J.P., Williams, S.R., Asprodini, E., and Crunelli, V. (1997). Postnatal development of membrane properties and delta oscillations in thalamocortical neurons of the cat dorsal lateral geniculate nucleus. J. Neurosci. *17*, 5428-5444.

Steriade, M., Contreras, D., Curró Dossi, R., and Nuñez, A. (1993). The slow (< 1 Hz) oscillation in reticular thalamic and thalamocortical neurons: scenario of sleep rhythm generation in interacting thalamic and neocortical networks. J. Neurosci. *13*, 3284-3299.

Williams SR, Turner JP, Anderson, C.M., and Crunelli, V. (1996). Electrophysiological and morphological properties of interneurones in the rat dorsal lateral geniculate nucleus in vitro. J. Physiol. (Lond) *490*, 129-147.

Xiang, Z., Huguenard, J.R., and Prince, D.A. (1998). Cholinergic switching within neocortical inhibitory networks. Science *281*, 985-988.

Zhu, J.J., Lytton, W.W., and Uhlrich, D.J. (1999a). An intrinsic oscillation in interneurons of the rat lateral geniculate nucleus. J. Neurophysiol. *81*, 702-711.

Zhu, J.J., Uhlrich, D.J., and Lytton, W.W. (1999b). Burst firing in identified rat geniculate interneurons. Neuroscience *91*, 1445-1460.

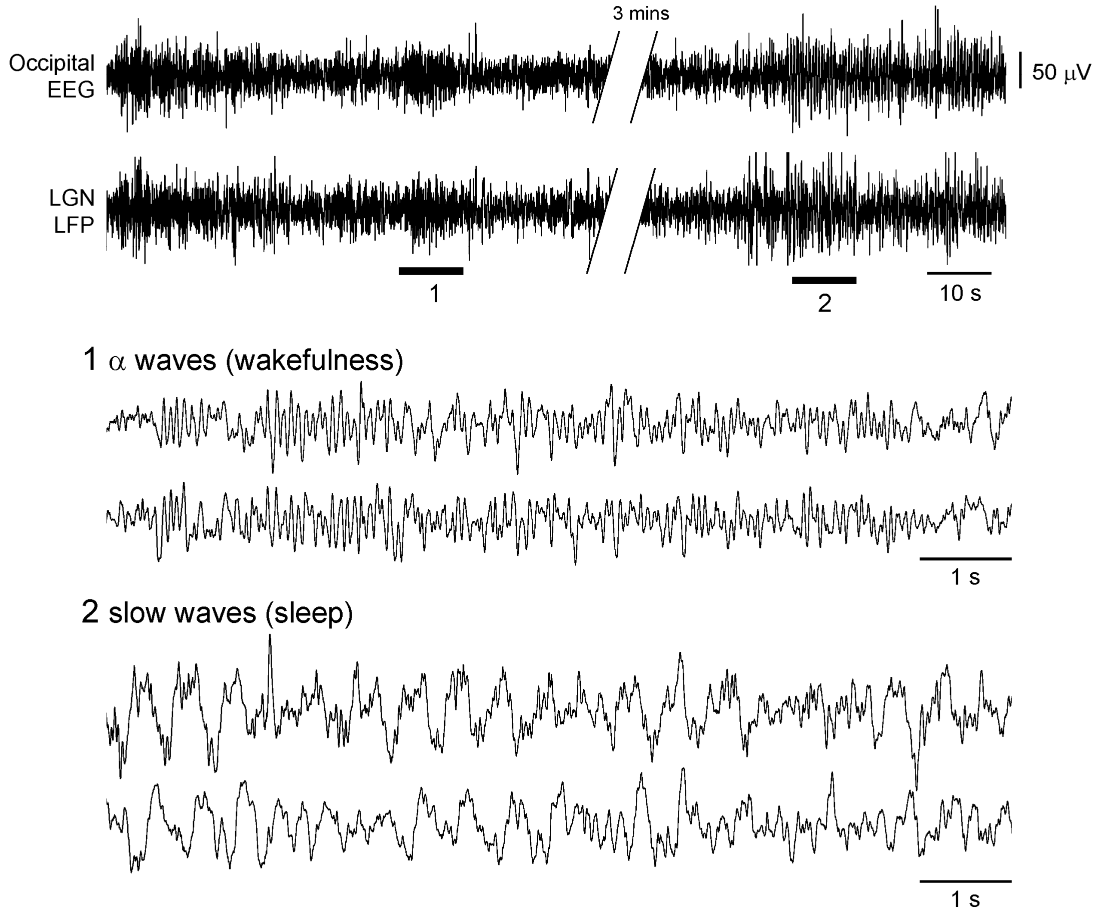

Supplementary Figure 1. Additional information regarding EEG and LFO recording in behaving cats.

Extended recording of the raw occipital EEG and thalamic LFP from the sites where the data in Fig. 1 was obtained. The underlined sections marked 1 and 2 are enlarged below and illustrate a period of α activity during when the animal was awake (1) and a period of slow waves when the animal was sleeping (2).

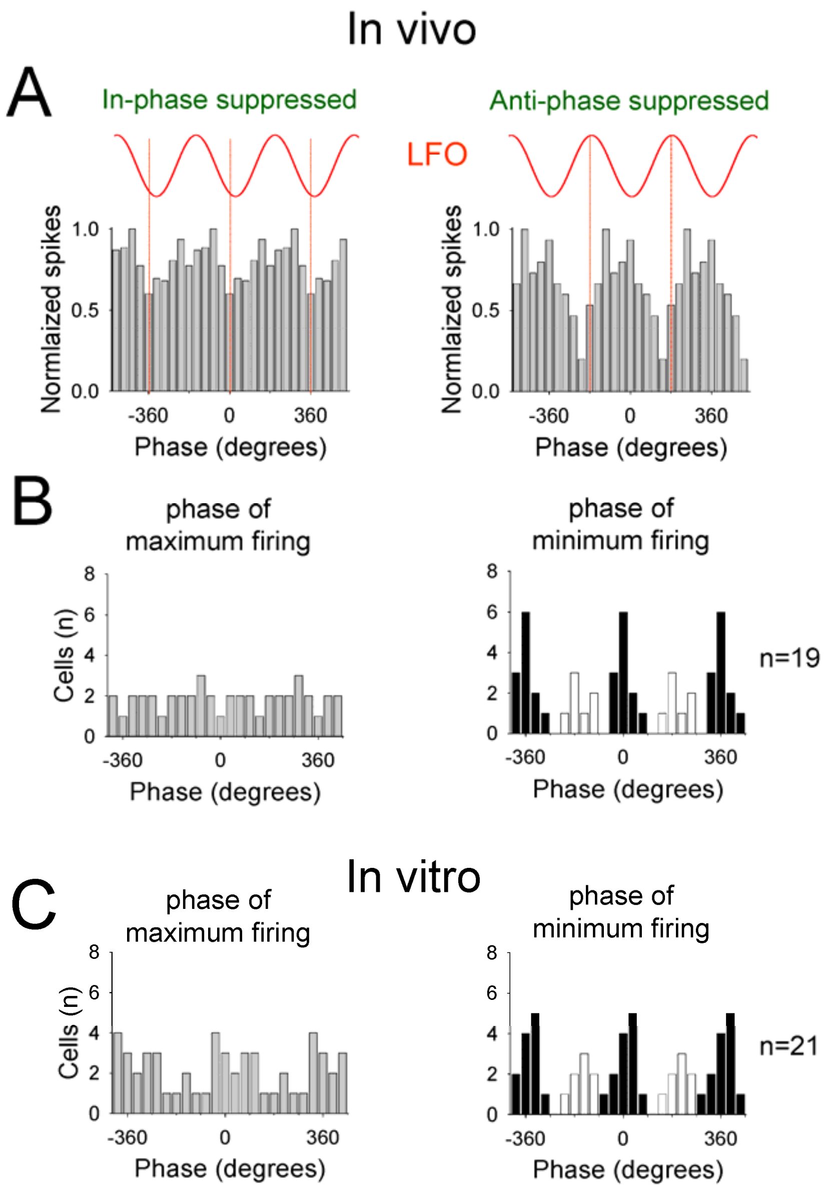

Supplementary Figure 2. Additional information relating to *in vivo* and *in vitro* recordings of relay-mode TC neurons.

(A) Examples of spike timing histograms from additional relay-mode LGN TC neurons recorded *in vivo* that reveal an in-phase (left) and anti-phase (right) suppressed output but which show distinct phases of the α rhythm at which peak firing takes place to the corresponding examples in Fig. 2A.

(B) Plot of the phases of the LFO at which the maximum (left) and minimum (right) amount of firing occur in individual relay-mode TC neurons *in vivo* reveals no consistent pattern in the former but a clear partitioning of the latter into clusters around the negative (black bars) and positive (white bars) LFO peaks (bin width 36° for each plot).

(C) Plot of the phases of the LFO at which the maximum (left) and minimum (right) amount of firing occur in individual relay-mode TC neurons *in vitro* shows a similar pattern to that observed *in vivo*.

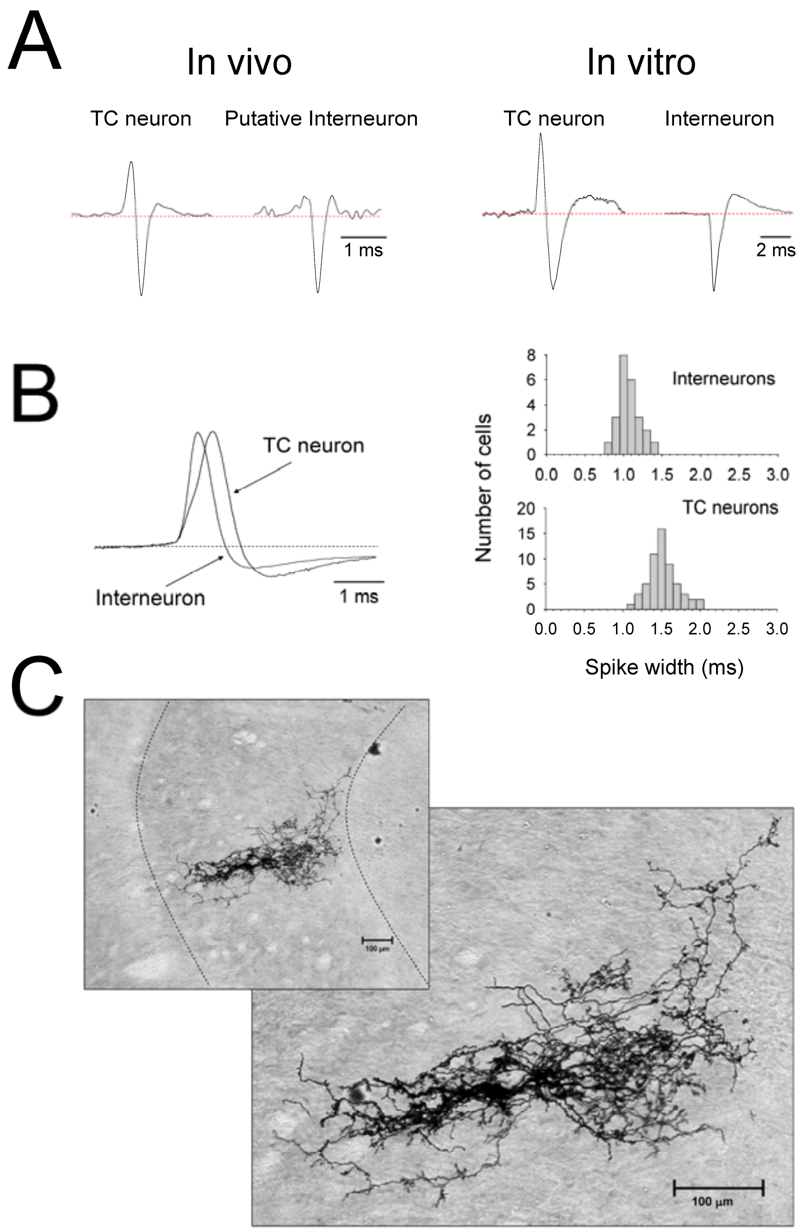

Supplementary Figure 3. Identification and additional details regarding LGN interneurons.

(A) Left panel: averaged unfiltered extracellular spike waveforms (10 spikes) for a TC neuron (left) and putative interneuron (right) recorded *in vivo*. Right panel: averaged unfiltered extracellular spike waveforms (10 spikes) for a TC neuron (left) and interneuron (right) recorded *in vitro*.

(B) Overlay of individual action potentials from an interneuron and TC neuron, recorded intracellularly *in vitro*, showing clear differences in their respective waveforms. This is further illustrated by the histograms on the right which show that the two populations of cells have distinct spike-width distributions.

(C) Morphology of an LGN interneuron recorded from lamina A *in vitro*. Note the typical extensive dendritic structure and the characteristic perpendicular orientation of the long axis of the cell with respect to the lamina borders (dashed lines). As with this cell, all inteneurons recorded *in vitro* were located in the centre of lamina A.

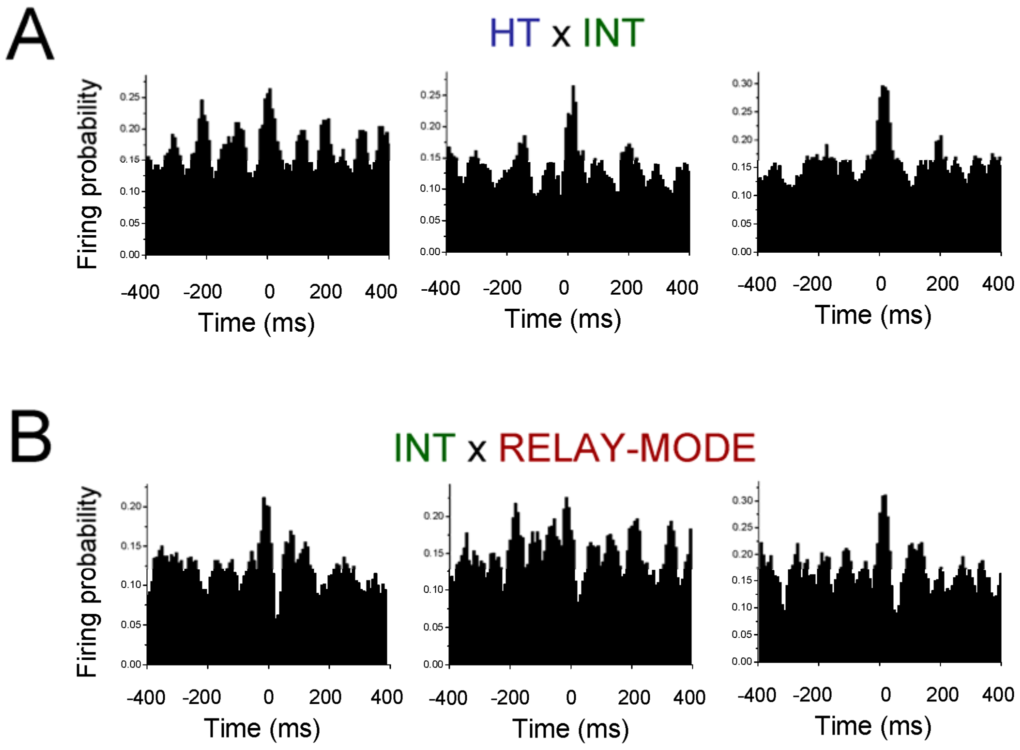

Supplementary Figure 4. Individual cross-correlograms for distinct HT bursting TC neuron-interneuron pairs and interneuron-relay-mode TC neuron pairs recorded in naturally waking cats.
(A) Cross-correlograms for 3 additional distinct HT bursting TC neuron-interneuron pairs (see Fig. 3D).
(B) Cross-correlograms for 3 additional distinct interneuron-relay-mode firing TC neuron pairs (see Fig. 3D).

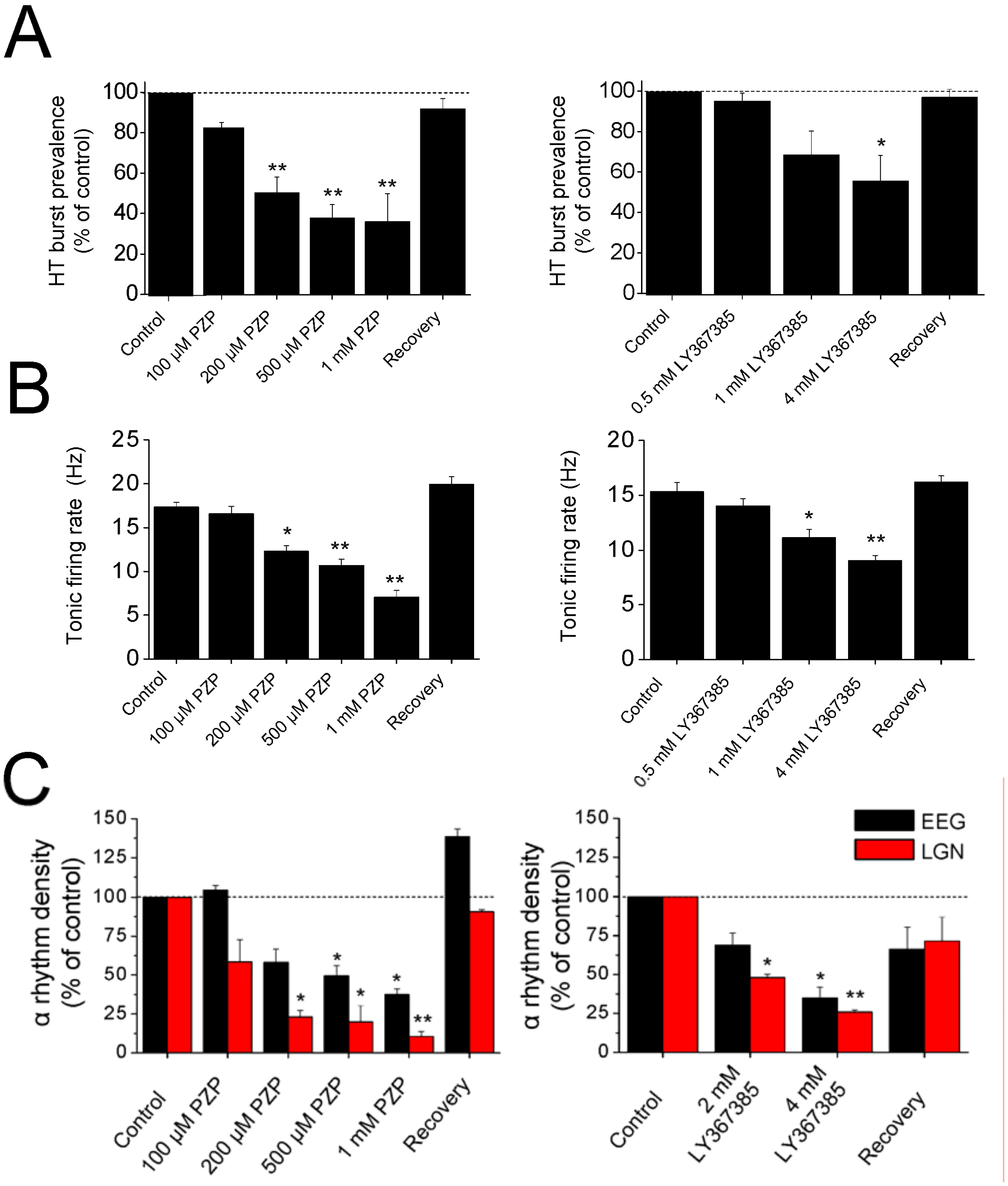

Supplementary Figure 5. Effects of thalamic reverse microdialysis of mAchR and mGluR1a antagonists on EEG and LGN α rhythm density and associated HT bursting and tonic firing/relay-mode in LGN TC neurons in naturally waking cats.
(A and B). Histograms summarizing the effect of thalamic application of pirenzipine (mAchR antagonist) (left) and LY367385 (mGluR1a antagonist) (right) on HT burst prevalence and tonic firing rate in LGN TC neurons (see Supp. table 1).
(C) Histograms summarizing the effect of thalamic pirenzipine (left) and LY367385 (right) application on EEG (black bars) and LGN (red bars) α rhythm density (see Supp. table 2).

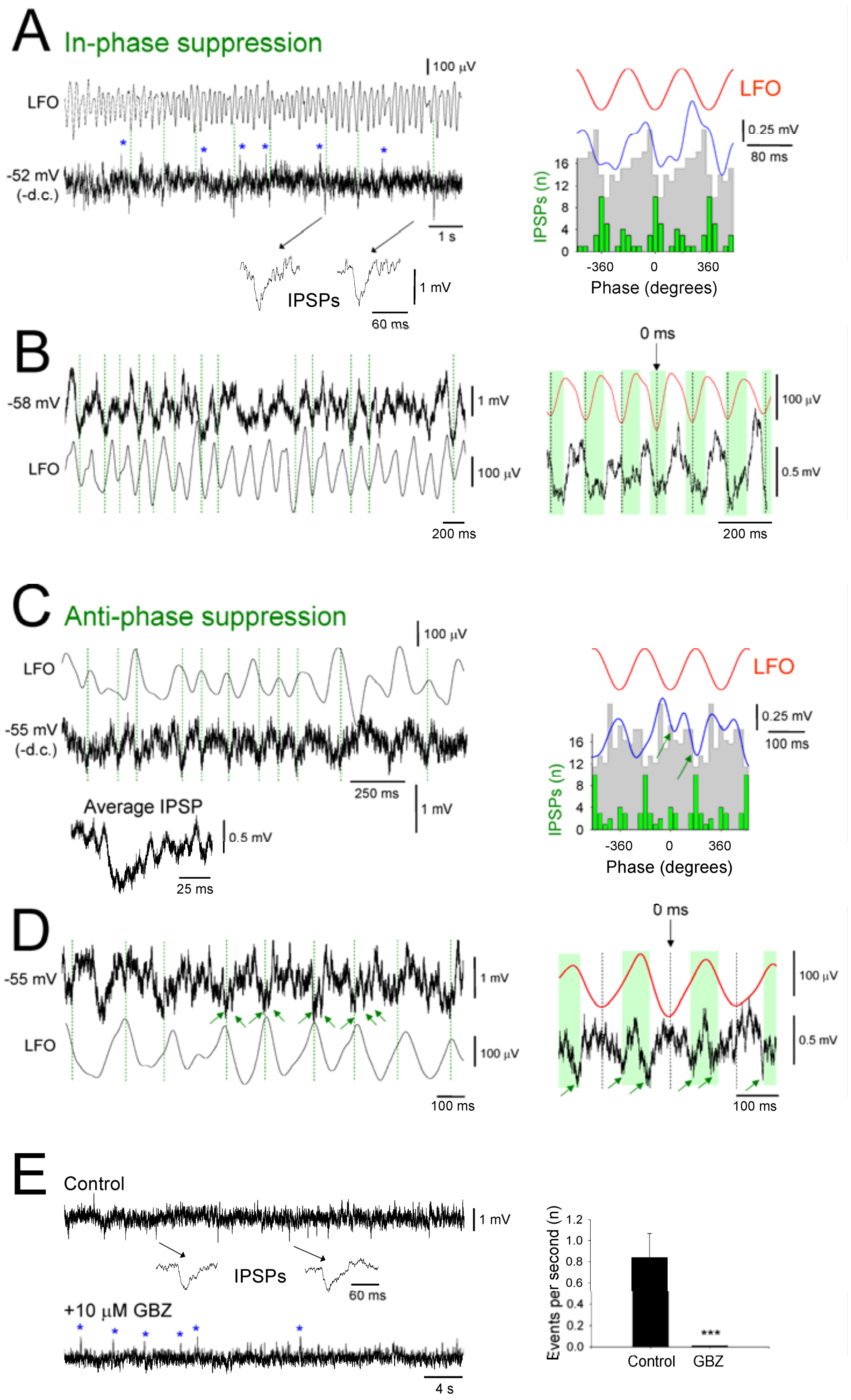
A
In-phase suppression
100 µV
LFO
-52 mV
(-d.c.)
1 s
IPSPs
1 mV
60 ms
LFO
0.25 mV
80 ms
IPSPs (n)
16
12
8
4
0
-360
0
360
Phase (degrees)
B
-58 mV
1 mV
LFO
100 µV
200 ms
0 ms
100 µV
0.5 mV
200 ms
C
Anti-phase suppression
100 µV
LFO
-55 mV
(-d.c.)
250 ms
1 mV
Average IPSP
0.5 mV
25 ms
LFO
0.25 mV
100 ms
IPSPs (n)
16
12
8
4
0
-360
0
360
Phase (degrees)
D
-55 mV
1 mV
LFO
100 µV
100 ms
0 ms
100 µV
0.5 mV
100 ms
E
Control
1 mV
IPSPs
60 ms
+10 µM GBZ
4 s
Events per second (n)
1.2
1.0
0.8
0.6
0.4
0.2
0.0
***
Control
GBZ

Supplementary Figure 6. The subthreshold membrane potential of relay-mode LGN TC neurons is sculpted by phasic inhibition during *in vitro* α rhythms.

(A) Simultaneous recording of the subthreshold membrane potential (bottom) of the in-phase suppressed relay-mode LGN TC neuron shown in the top of Fig. 2C and the proximal LFO (top). Note the occurrence of sporadic IPSPs, two of which are enlarged below. Note also the presence of occasional brief depolarizing events (blue asterisks; see Supp. results section C and Supp. Fig. 7 for further details). The plot to the right shows that IPSPs predominantly occur near the negative LFO peak (green bars). The blue trace is the LFO-triggered subthreshold membrane potential average filtered at 2-20 Hz which shows an essentially monophasic form, i.e. a single peak and trough during each oscillation cycle. The grey shading represents the spike timing (see Fig. 2C).

(B) Subthreshold membrane potential recording from another in-phase suppressed relay-mode TC neuron *in vitro* showing IPSPs that mainly occur in-phase with the negative LFO peaks (dotted green lines). The corresponding LFO-triggered membrane potential average is shown to the right and, again, shows an essentially monophasic appearance whereby each oscillation cycle consists of a single inhibitory (green shaded area) and excitatory phase.

(C) Simultaneous recording of the subthreshold membrane potential (bottom) of the anti-phase suppressed relay-mode LGN TC neuron shown in the bottom of Fig. 2C and the proximal LFO (top). Again, note the presence of IPSPs, the average of which is shown below. The plot to the right shows that in this cell these events primarily occur close to the positive LFO peak (green bars). The blue trace is the LFO-triggered subthreshold membrane potential average filtered at 2-20 Hz which shows two clear troughs during the first positive LFO wave (green arrows). Again, the grey shading represents the spike timing (see Fig. 2C).

(D) Subthreshold membrane potential recording from an additional anti-phase suppressed relay-mode TC neuron *in vitro* showing IPSPs that mainly occur in-phase with the positive LFO peaks (dotted green lines). Note the presence of multiple events during some oscillation cycles (green arrows). The corresponding subthreshold membrane potential average is shown to the right and shows a biphasic appearance whereby the positive phase of each LFO cycle consists of two brief inhibitory excursions (green shaded area).

(E) Left: subthreshold membrane potential recording from a relay-mode TC neuron in the presence of 50 μM Cch *in vitro* showing IPSPs (top). Application of 10 μM GBZ abolishes these events confirming that they are genuine GABAergic IPSPs and increases the occurrence of depolarizing events (bottom; see Supp. results section C and Supp. Fig. 7 for further details). Right: summary of the effect of 10 μM GBZ on IPSP generation in relay-mode TC neurons (n=10).

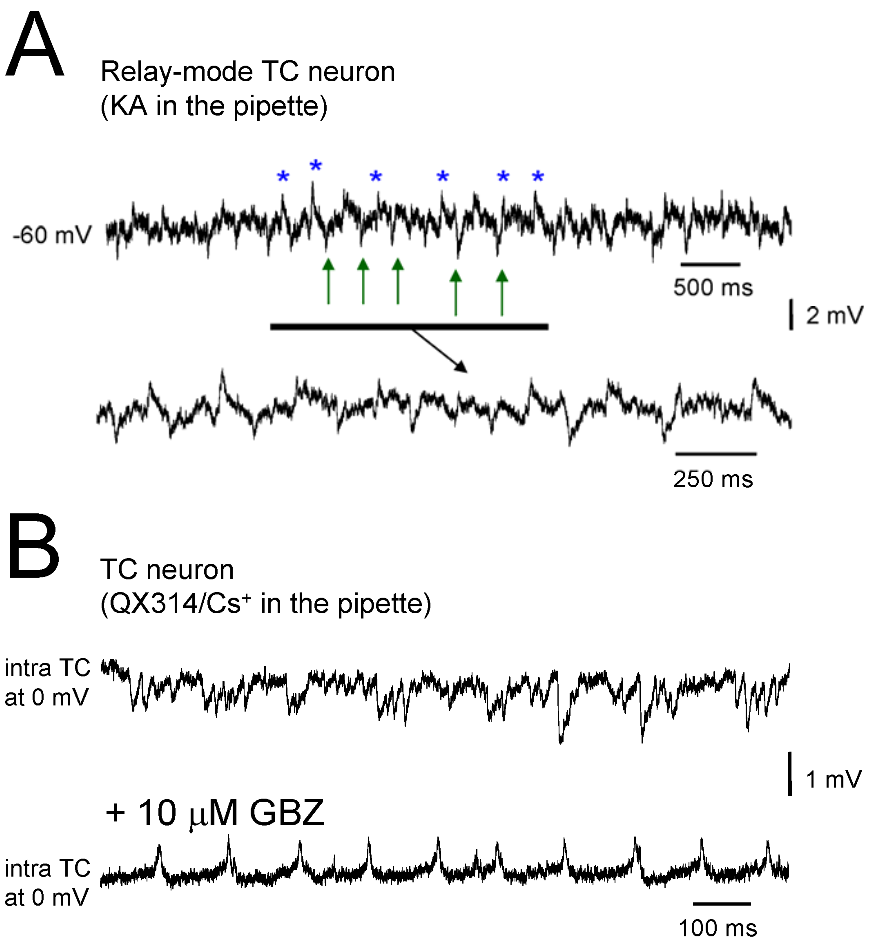

Supplementary Figure 7. Evidence that some relay-mode TC neurons exhibit weak GJ-mediated connections with the HT bursting TC neuron network.

(A) Relay-mode TC neuron recorded in the presence of 50 µM Cch using a KAc-filled electrode showing both rhythmic IPSPs (green arrows) and small amplitude, fast depolarizing events (blue asterisks). The underlined section is enlarged below as indicated. See also Supp. Fig. 6A and E.

(B) TC neuron recorded at 0 mV in the presence of 50 µM Cch using a Cs^+/QX314-filled electrode showing rhythmic IPSP bursts (cf. Fig. 5D). In this neuron application of 10 µM GBZ blocks the IPSPs and reveals rhythmic burstlets.

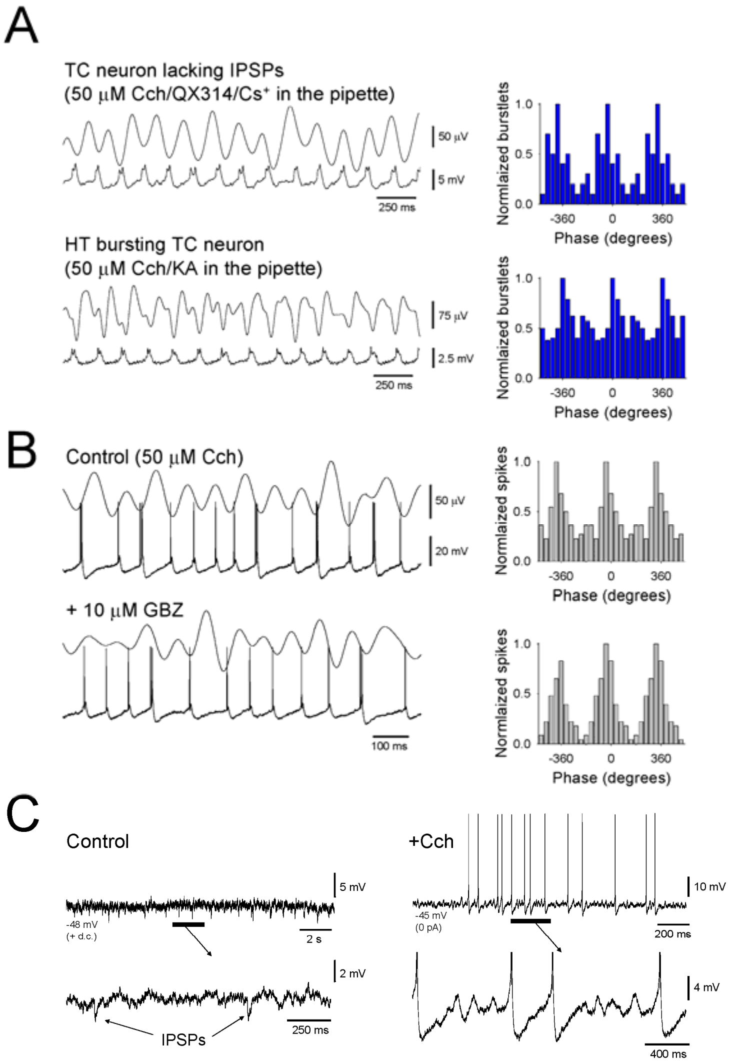

Supplementary Figure 8. Additional information regarding HT bursting TC neurons recording in the presence of 50 μM Cch *in vitro*.

(A) Top: TC neuron recorded at 0 mV in the presence of 50 μM Cch using a Cs^+/QX314-filled electrode showing rhythmic burstlets that are correlated with the negative peak of the simultaneously-acquired LFO (see burstlet timing histogram shown on the right). Bottom: these burstlets are essentially indistinguishable from those recorded from HT bursting TC neurons using KAc-filled electrodes. The corresponding burstlet timing histogram is shown on the right.

(B) Top: HT bursting TC neuron recorded in the LGN slice in the presence of 50 μM Cch where action potential output is clearly correlated with the negative peak of the simultaneously-acquired LFO (see spike timing histogram on the right). Bottom: this neuron remains robustly correlated with the LFO following application of 10 μM GBZ. Again, the corresponding spike timing histogram is shown to the right.

(C) HT bursting TC neuron in the LGN slice recorded in control conditions showing occasional IPSPs as indicated. Application of 50 μM Cch brings about spontaneous bursting and abolishes IPSPs.

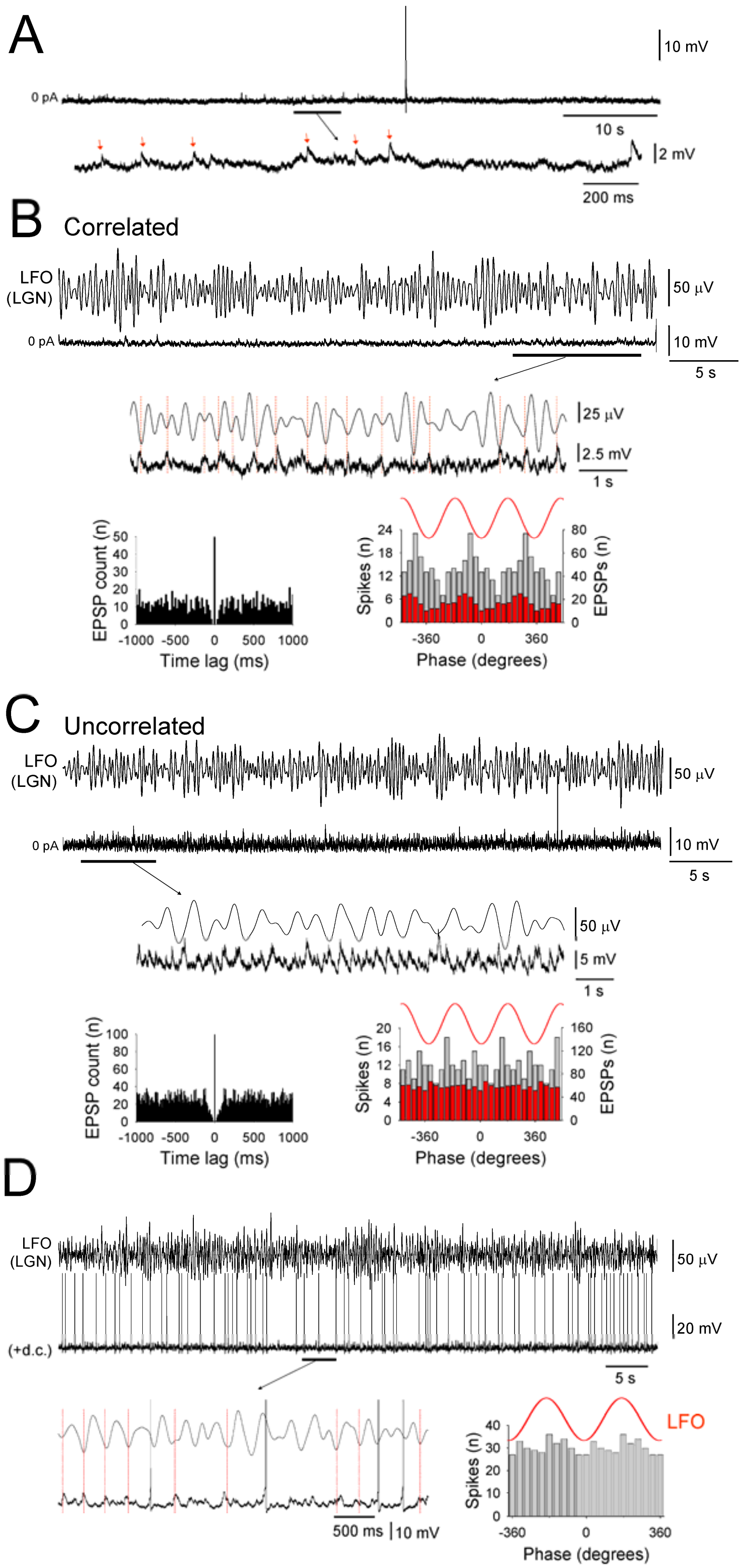
A
10 mV
0 pA
10 s
2 mV
200 ms
B Correlated
LFO (LGN)
50 µV
0 pA
10 mV
5 s
25 µV
2.5 mV
1 s
EPSP count (n)
Time lag (ms)
Spikes (n)
EPSPs (n)
Phase (degrees)
C Uncorrelated
LFO (LGN)
50 µV
0 pA
10 mV
5 s
50 µV
5 mV
1 s
EPSP count (n)
Time lag (ms)
Spikes (n)
EPSPs (n)
Phase (degrees)
D
LFO (LGN)
50 µV
20 mV
(+d.c.)
5 s
500 ms
10 mV
LFO
Spikes (n)
Phase (degrees)

Supplementary Figure 9. PGN neuron firing during *in vitro* α rhythms is sparse and largely uncorrelated with the ongoing LFO in the LGN.

(A) Intracellular recording of a PGN neuron in the absence of any steady current showing sparse action potential output in the presence of 50 μM Cch. The underlined section is enlarged below and shows a group of EPSPs (red arrows).

(B) Simultaneous recording of PGN neuron activity where subthreshold excitatory input was significantly correlated with LGN α activity. The underlined section is enlarged below as indicated and shows the correspondence between EPSPs and the negative LFO peaks. Shown further below on the left is the corresponding auto-correlogram illustrating a lack of rhythmicity in the EPSPs. Shown on the bottom right is the EPSP timing histogram which confirms a predilection for EPSPs to occur close to the negative LFO peak (red bars), and spike timing histogram corresponding to when the neuron is subject to steady depolarization (grey bars) which reveals that under this condition action potentials also occur mainly near the negative LFO peak (but see also D).

(C) Simultaneous recording of PGN neuron activity and the proximal LFO in the adjacent LGN for a PGN cell where subthreshold excitatory input was not correlated with LGN α activity. The underlined section is enlarged below as indicated and reveals prominent excitatory synaptic input. Shown further below on the left is the corresponding auto-correlogram illustrating a lack of rhythmicity in the EPSPs. Shown on the bottom right is the EPSP timing histogram which confirms that despite being extensive, synaptic activity in this neuron does not correlate with the LFO (red bars). Accordingly, the spike timing histogram (grey bars) shows that when the neuron is subject to steady depolarization action potentials also do not correlate with the LFO.

(D) Simultaneous recording of a PGN neuron, in the presence of steady depolarizing current to bring about firing, where spontaneous subthreshold excitatory input was significantly correlated with LGN α activity. However, unlike the neuron shown in panel B, the spike timing histogram, which is shown to the right, reveals that in this cell even though subthreshold activity is correlated, action potential output is not.

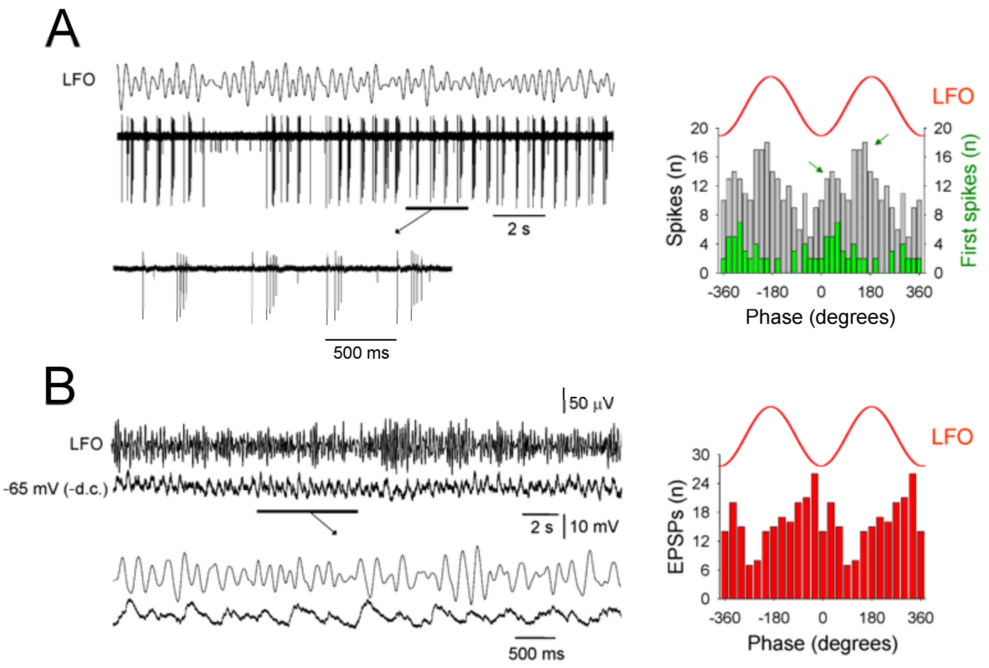

Supplementary Figure 10. Additional information regarding the activity of LGN interneurons recorded *in vitro*.

(A) Extracellular recording of the spontaneous activity of an interneuron which can exhibit up to 10 spikes in an individual burst and which is associated with a biphasic spike timing histogram (right, see arrows) that is similar to that shown in the bottom of Fig. 6B. This is again due to the large interval between the first (green bars) and second spike in each burst. The unfiltered average spike waveform from this recording is shown in Supp. Fig. 3A (right).

(B) Rhythmic EPSP complexes from the interneuron shown in Fig. 6B. The underlined section is expanded below as indicated. The EPSP timing histogram is shown to the right and indicates that EPSPs in this cell mainly occur close to the negative LFO peak.

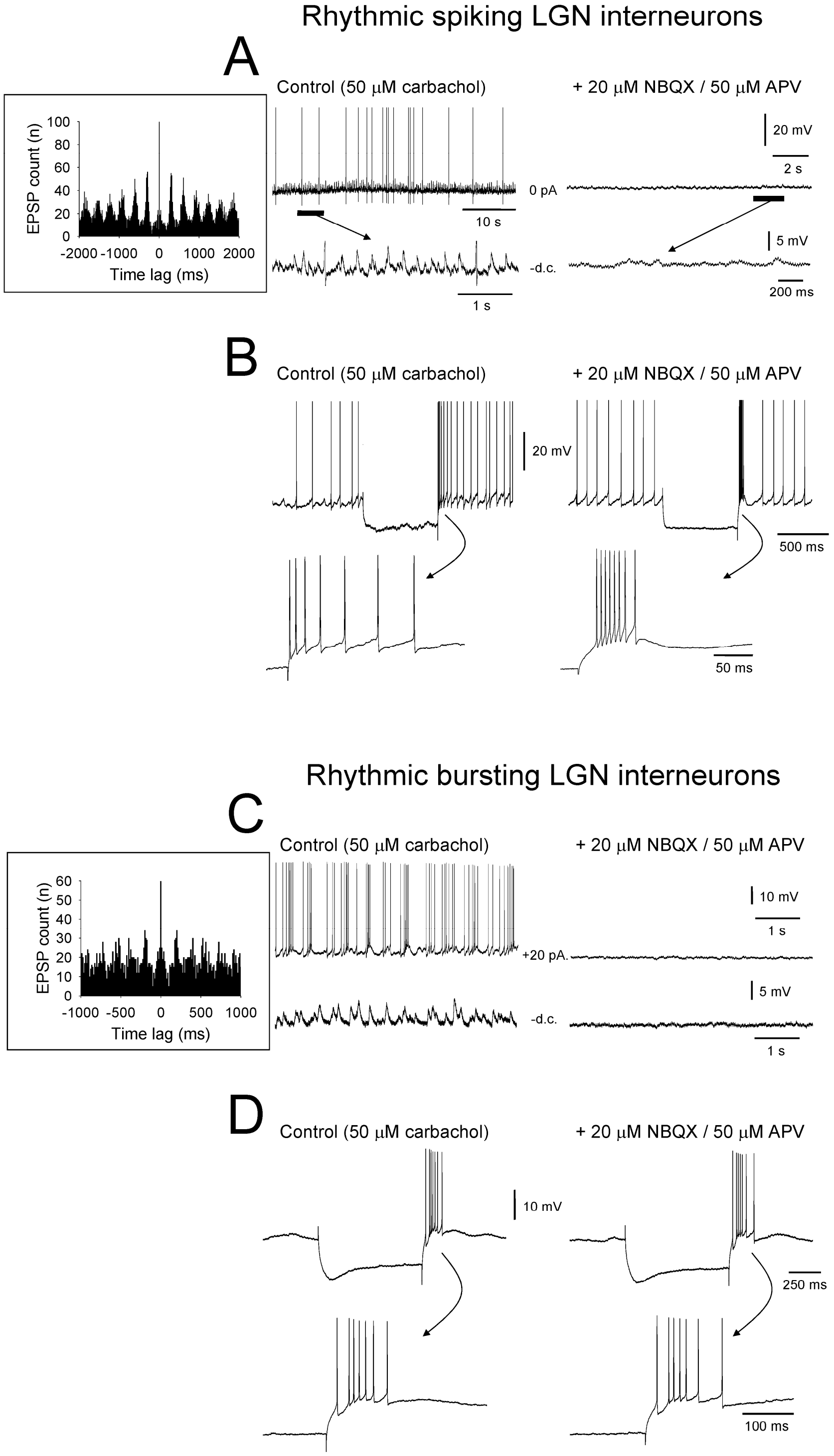

Rhythmic spiking LGN interneurons
A
Control (50 µM carbachol)
+ 20 µM NBQX / 50 µM APV
EPSP count (n)
100
80
60
40
20
0
-2000 -1000 0 1000 2000
Time lag (ms)
20 mV
2 s
0 pA
10 s
5 mV
-d.c.
1 s
200 ms
B
Control (50 µM carbachol)
+ 20 µM NBQX / 50 µM APV
20 mV
500 ms
50 ms
Rhythmic bursting LGN interneurons
C
Control (50 µM carbachol)
+ 20 µM NBQX / 50 µM APV
EPSP count (n)
60
50
40
30
20
10
0
-1000 -500 0 500 1000
Time lag (ms)
10 mV
1 s
+20 pA
5 mV
-d.c.
1 s
D
Control (50 µM carbachol)
+ 20 µM NBQX / 50 µM APV
10 mV
250 ms
100 ms

Supplementary Figure 11. Reliance of interneuron output on a rhythmic excitatory drive in the presence of Cch and the intrinsic nature of bursting in LGN interneurons *in vitro*.

(A) Intracellular recording of an LGN interneuron in the presence of 50 μM Cch showing spontaneous action potential output in the absence of steady current (top left) and rhythmic EPSPs when hyperpolarized (bottom left; see also auto-correlogram in the inset to the far left). Blockade of excitatory synaptic transmission with NBQX and APV abolishes both spontaneous firing (top right) and rhythmic EPSPs (bottom right).

(B) Response of an LGN interneuron that is unable to exhibit rhythmic bursting in the presence of 50 μM Cch to a brief negative current step. Note the absence of prominent burst firing at the offset of the current step (enlarged below as indicated). The same current step given following application NBQX and APV leads to a strong burst.

(C) Intracellular recording of an LGN interneuron in the presence of 50 μM Cch showing repetitive burst firing following injection of a small amount of steady depolarizing current and rhythmic EPSPs when hyperpolarized (bottom left; see also auto-correlogram in the inset to the far left). Blockade of excitatory synaptic transmission with NBQX and APV abolishes both spontaneous firing (top right) and rhythmic EPSPs (bottom right).

(D) Response of an LGN interneuron that is able to exhibit rhythmic bursting in the presence of 50 μM Cch to a brief negative current step. Note the presence of prominent burst firing at the offset of the current step (enlarged below as indicated). This burst response is unaffected by NBQX/APV confirming its intrinsic nature.

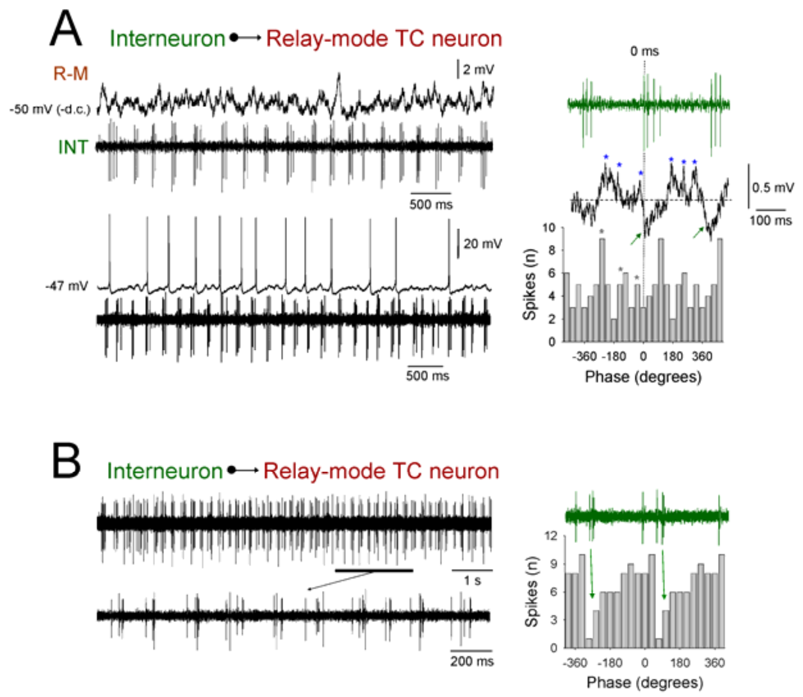

Supplementary Figure 12. Relay-mode TC neurons show an inhibition and suppression of firing coincident with interneuron output *in vitro*.

(A) Top: simultaneous intracellular recording of a relay-mode (R-M) TC neuron in the presence of -100 pA steady hyperpolarizing current and extracellular recording of a closely situated interneuron. Bottom: same recordings but with the relay-mode TC neuron not subject to steady hyperpolarizing current. Shown to the right is the average subthreshold activity of the relay-mode TC neuron (black trace, calculated from the top left traces) triggered by the first spike in an interneuron burst (shown above in green for reference purposes). The spike timing histogram is for the relay-mode TC neuron relative to interneuron burst generation (constructed from the bottom set of traces). Note the presence of IPSPs (green arrows) in synchrony with the interneuron bursts, their precession by burstlets (spikelets indicated by blue asterisks) and the general correspondence between subthreshold input and suprathreshold activity (grey asterisks mark points of approximate correspondence between average spikelets and elevations in firing).

(B) Extracellular double unit recording from the LGN slice comprising the activity of an interneuron (smaller amplitude unit) and relay-mode TC neuron (larger amplitude unit). The underlined section is expanded below. The spike timing histogram to the right is for the relay-mode TC neuron relative to interneuron burst generation (constructed such that the midpoint of the interneuron bursts represent 0 and 360 degrees) and illustrates a strong suppression of action potential output toward the end of the interneuron bursts (green arrows) (cf. Fig. 3D, right). The green trace above is a section of interneuron bursting taken from the same recording and is provided for illustrative purposes. Note that in panels A and B of this figure the single unit recordings have been band-pass filtered (see experimental procedures) leading to an alteration of the original extracellular spike waveform (see Supp. Fig. 3A).

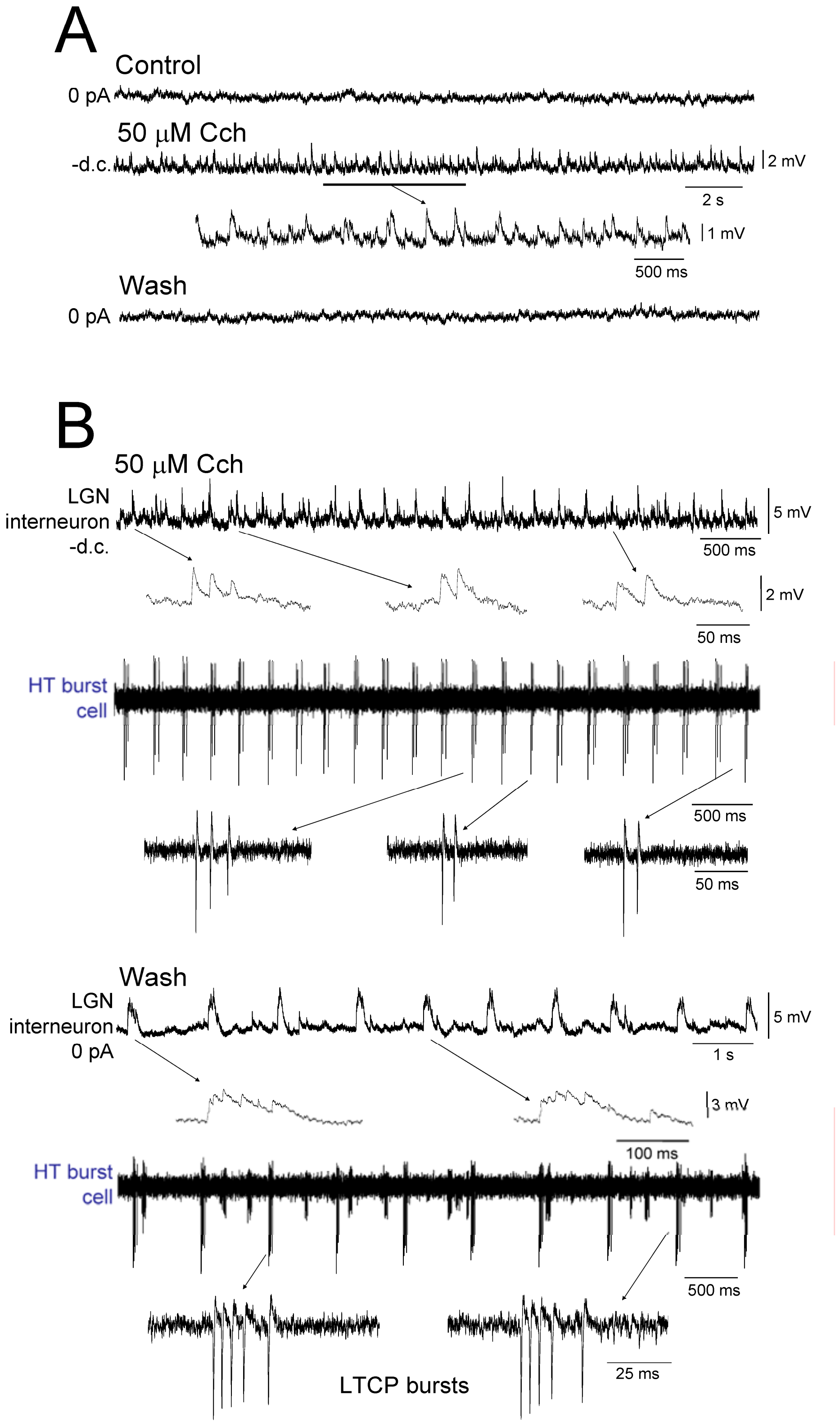
A
Control
0 pA
50 μM Cch
-d.c.
2 mV
2 s
1 mV
500 ms
Wash
0 pA
B
50 μM Cch
LGN interneuron -d.c.
5 mV
500 ms
2 mV
50 ms
HT burst cell
500 ms
50 ms
Wash
LGN interneuron 0 pA
5 mV
1 s
3 mV
100 ms
HT burst cell
500 ms
25 ms
LTCP bursts

Supplementary Figure 13. Dynamic regulation of excitatory synaptic events in LGN intereneurons by cholinergic input *in vitro*.

(A) Intracellular recording of an LGN interneuron showing the reversible instatement of rhythmic EPSP complexes following 50 μM Cch application.

(B) Top traces, 50 μM Cch: LGN interneuron recorded in the presence of 50 μM Cch showing rhythmic EPSP complexes when hyperpolarized with steady current (see enlargements as indicated). Shown below is an extracellular recording of an HT bursting TC neuron also in the presence of 50 μM Cch. Individual bursts are enlarged as indicated and reveal a spike pattern which broadly matches the EPSP complexes in the interneuron recording above. Bottom traces, Wash: following washout of Cch the same LGN interneuron exhibits a distinct type of EPSP complex which occurs at a lower frequency. Specifically, these comprise a series of EPSPs that gradually get further apart as the complex progresses (see enlargements as indicated). Shown below is an extracellular recording of the same HT bursting TC neuron depicted in the top set of traces, again following washout of Cch. This neuron now exhibits unmistakable LTCP-mediated bursts (see enlargements as indicated) that have a characteristic decelerating pattern of action potential output that broadly matches the distinct type of EPSP complexes shown above.

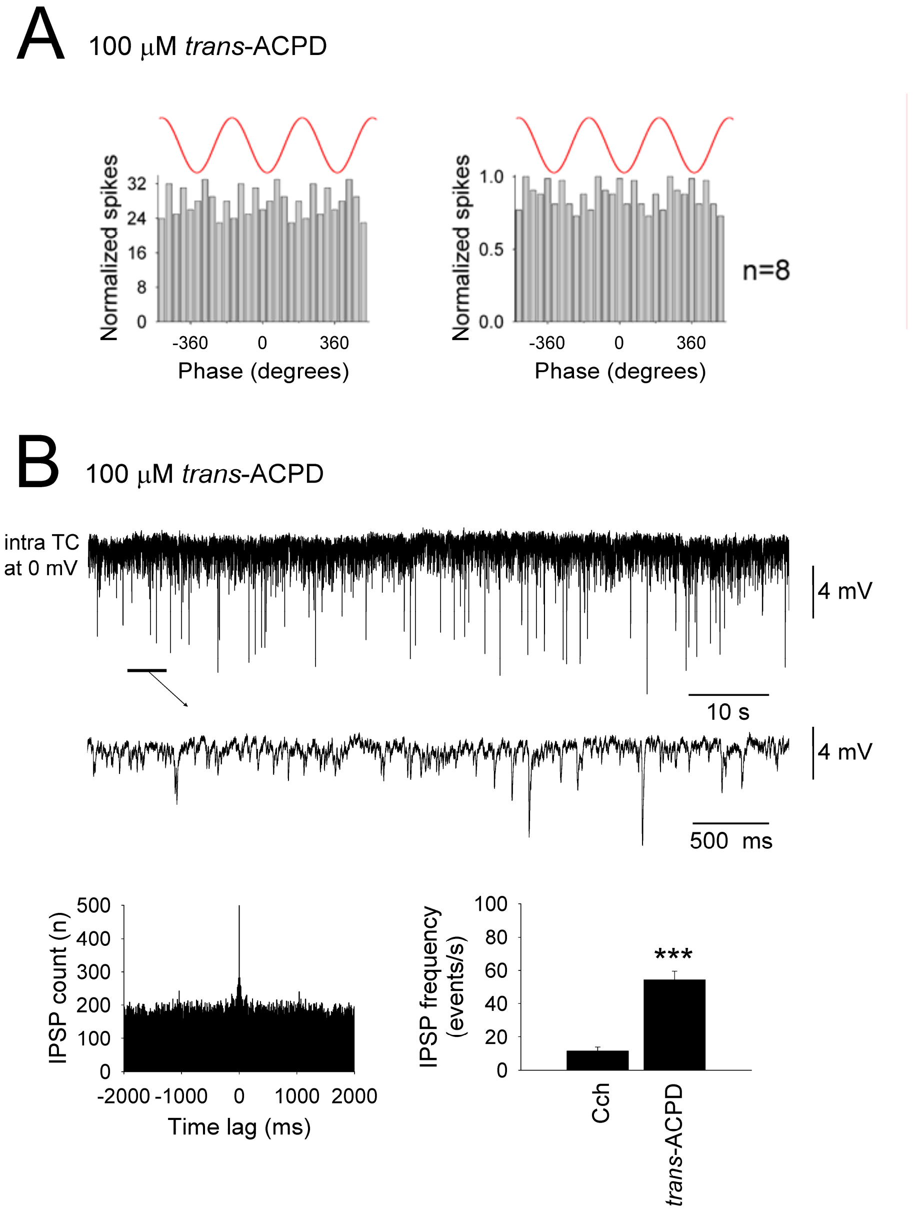

Supplementary Figure 14. Lack of temporal framing in LGN TC neurons following *trans*-ACPD application *in vitro* is due to indiscriminate inhibition.

(A) Spike timing histograms for a single relay-mode TC neuron (left) and for 8 relay-mode TC neurons (right) recorded during α rhythms that has been brought about by *trans*-ACPD application (see Hughes et al., 2004) showing that in this condition there is no correlation between this form of firing and ongoing α activity (i.e. lack of temporal framing).

(B) Top traces: recordings of relay-mode firing TC neurons using Cs^+/QX314-filled electrodes reveals that the lack of temporal framing in these cells following *trans*-ACPD application is due to non-rhythmic indiscriminate inhibition. Bottom left: auto-correlogram confirming a lack of rhythmicity in IPSPs from the neuron depicted above. Bottom right: histogram summarizing the mean frequency of IPSPs in relay-mode TC neurons recorded in the presence of 100 μM *trans*-ACPD or 50 μM Cch. (***, $p<0.001$)

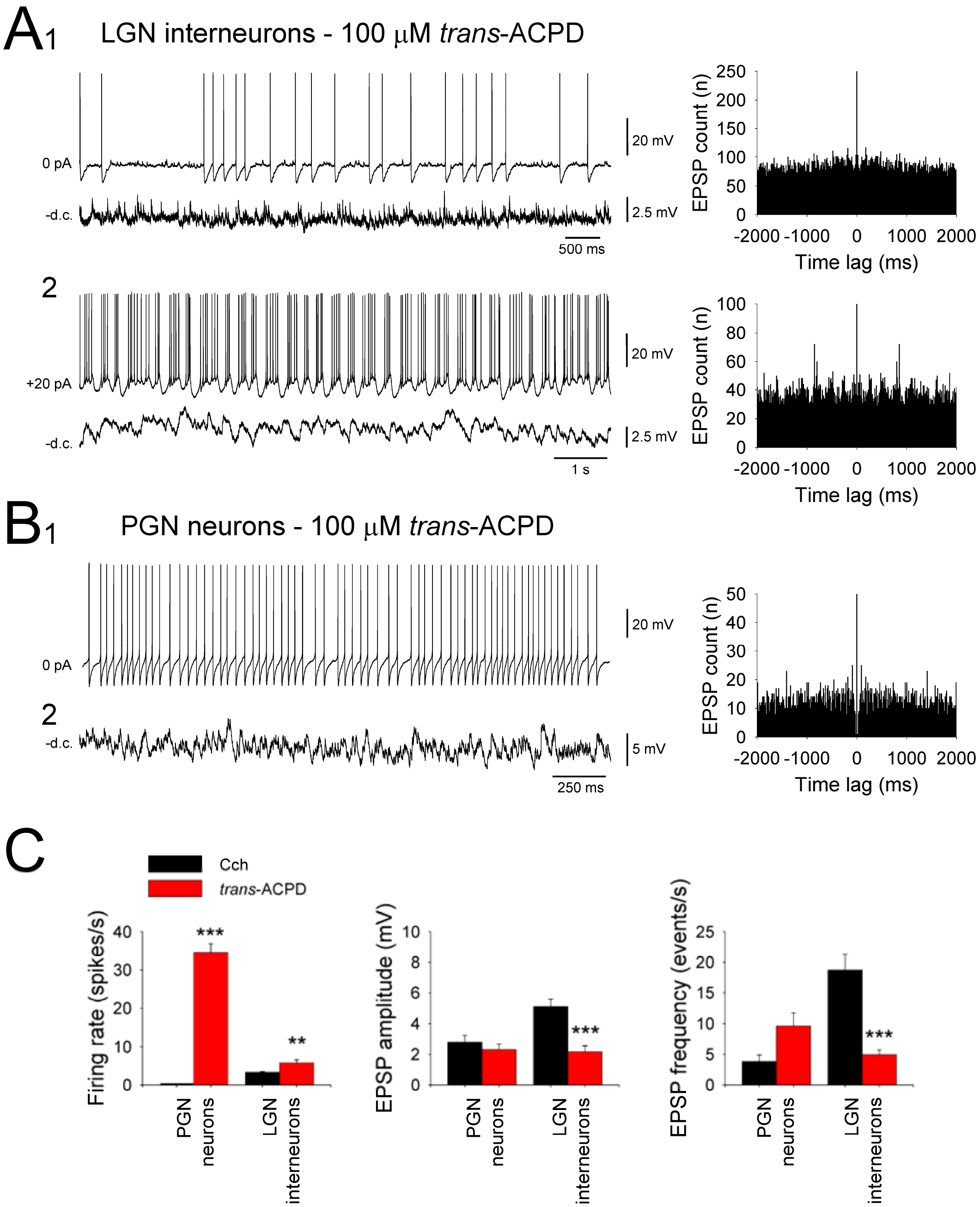

A1 LGN interneurons - 100 μM trans-ACPD
0 pA
-d.c.
20 mV
2.5 mV
500 ms
EPSP count (n)
Time lag (ms)
2
+20 pA
-d.c.
20 mV
2.5 mV
1 s
B1 PGN neurons - 100 μM trans-ACPD
0 pA
2
-d.c.
20 mV
5 mV
250 ms
C
Cch
trans-ACPD
Firing rate (spikes/s)
EPSP amplitude (mV)
EPSP frequency (events/s)
PGN neurons
LGN interneurons
***
**

Supplementary Figure 15. Activity of LGN interneurons and PGN neurons in the presence of *trans*-ACPD *in vitro*.

(A) Top traces, 1: intracellular recording of an LGN interneuron obtained following *trans*-ACPD application showing spontaneous action potential output in the absence of any steady current (top). Injection of steady hyperpolarizing current reveals prominent excitatory synaptic input (bottom) that is non–rhythmic (see auto-correlogram to the far right). Bottom traces, 2: intracellular recording of a different LGN interneuron obtained in the presence of *trans*-ACPD showing repetitive burst firing following injection of a small amount of steady depolarizing current (top). Again, hyperpolarization of this neuron reveals prominent excitatory synaptic input (bottom) which is also essentially non–rhythmic (see auto-correlogram to the far right).

(B) Top trace, 1: intracellular recording of a PGN neuron obtained following *trans*-ACPD application showing spontaneous high frequency (~30 Hz) action potential output in the absence of any steady current. Bottom trace, 2: intracellular recording of a different PGN neuron in the presence of *trans*-ACPD and following the injection of steady hyperpolarizing current again reveals prominent excitatory synaptic input which is non-rhythmic (see auto-correlogram to the far right).

(C) Histograms summarizing mean firing rate, EPSP amplitude and individual EPSP frequency in LGN interneurons and PGN neurons recorded in the presence of 50 μM Cch (black bars) and 100 μM *trans*-ACPD (red bars). (**, $p<0.01$; ***, $p<0.001$)

Supplementary Table 1. Effect of thalamic pirenzipine and LY367385 application on HT burst prevalence and tonic (i.e. relay-mode) firing rate in LGN TC neurons *in vivo*.

	HT burst prevalence (bursts per minute)	Tonic firing rate (Hz)
Control (n=4 neurons)	86.45±10.55	17.34±0.53
100 μM pirenzipine	73.6±13.75	16.56±0.86
200 μM pirenzipine	42.4±2.88**	12.3±0.63 Hz*
500 μM pirenzipine	32.2±5.32**	10.7±0.69**
1 mM pirenzipine	33.0±7.0**	7.02±0.77**
Wash	79.6±11.48	19.91±0.89
Control (n=3 neurons)	75.53±24.7	15.33±0.85
0.5 mM LY367385	73.4±24.43	14.04±0.66
1 mM LY367385	52.73±22.81	11.13±0.78*
2 mM LY367385	50.23±24.32	10.21±0.94*
4 mM LY367385	46.8±23.6*	9.06±0.46**
Wash	74.8±26.37	16.21±0.56

(*p<0.05; **p<0.01)

Supplementary Table 2. Effect of thalamic pirenzipine and LY367385 application on α rhythm density and power *in vivo*.

	LGN		EEG	
	Density (%)	Power (x 1000 μV^2)	Density (%)	Power (x 1000 μV^2)
Control (n=30)	39.34±5.34	3.87±0.51	32.95±7.09	7.22±1.00
100 μM pirenzipine	24.70±5.94	1.98±0.40	31.73±3.09	5.71±0.96
200 μM pirenzipine	11.29±1.83*	2.31±0.35	19.66±1.9	4.83±0.90
500 μM pirenzipine	7.13±2.84*	0.73±0.19**	16.5±2.1*	2.89±0.52**
1 mM pirenzipine	4.47±1.37**	0.54±0.08**	14.88±2.41*	2.44±0.38***
Wash	35.2±2.97	4.81±1.55	42.36±3.8	2.73±0.25
Control (n=30)	27.38±5.45	1.57±0.21	28.76±7.93	8.26±1.32
2 mM LY367385	22.3±5.13	0.86±0.10*	19.83±2.23	6.09±0.73
4 mM LY367385	13.16±4.81*	0.66±0.12*	10.06±1.97*	4.0±0.70*
Wash	19.58±4.2	1.55±0.37	19.08±4.05	8.57±1.04

(*p<0.05; **p<0.01; *** p<0.001)
